# Varied performance of picture description task as a screening tool across MCI subtypes

**DOI:** 10.1371/journal.pdig.0000197

**Published:** 2023-03-13

**Authors:** Joel A. Mefford, Zilong Zhao, Leah Heilier, Man Xu, Guifeng Zhou, Rachel Mace, Kelly L. Sloane, Shannon M. Sheppard, Shenly Glenn

**Affiliations:** 1 Department of Neurology, University of California, Los Angeles, California, United States of America; 2 Miro Health, Inc., San Francisco, California, United States of America; 3 Department of Neurology, Johns Hopkins University School of Medicine, Baltimore, Maryland, United States of America; 4 Department of Neurology, University of Pennsylvania, Philadelphia, Pennsylvania, United States of America; 5 Department of Communication Sciences & Disorders, Chapman University, Orange, California, United States of America; University of Bayreuth: Universitat Bayreuth, GERMANY

## Abstract

A picture description task is a component of Miro Health’s platform for self-administration of neurobehavioral assessments. Picture description has been used as a screening tool for identification of individuals with Alzheimer’s disease and mild cognitive impairment (MCI), but currently requires in-person administration and scoring by someone with access to and familiarity with a scoring rubric. The Miro Health implementation allows broader use of this assessment through self-administration and automated processing, analysis, and scoring to deliver clinically useful quantifications of the users’ speech production, vocal characteristics, and language. Picture description responses were collected from 62 healthy controls (HC), and 33 participants with MCI: 18 with amnestic MCI (aMCI) and 15 with non-amnestic MCI (naMCI). Speech and language features and contrasts between pairs of features were evaluated for differences in their distributions in the participant subgroups. Picture description features were selected and combined using penalized logistic regression to form risk scores for classification of HC versus MCI as well as HC versus specific MCI subtypes. A picture-description based risk score distinguishes MCI and HC with an area under the receiver operator curve (AUROC) of 0.74. When contrasting specific subtypes of MCI and HC, the classifiers have an AUROC of 0.88 for aMCI versus HC and and AUROC of 0.61 for naMCI versus HC. Tests of association of individual features or contrasts of pairs of features with HC versus aMCI identified 20 features with p-values below 5e-3 and False Discovery Rates (FDRs) at or below 0.113, and 61 contrasts with p-values below 5e-4 and FDRs at or below 0.132. Findings suggest that performance of picture description as a screening tool for MCI detection will vary greatly by MCI subtype or by the proportion of various subtypes in an undifferentiated MCI population.

## Author summary

Picture description has been used as a screening tool for identification of individuals with Alzheimer’s disease and mild cognitive impairment (MCI) and is a component of Miro Health’s platform for self-administration of neurobehavioral assessments. The Miro Health implementation allows broad use of this assessment through self-administration and automated processing, analysis, and scoring to deliver clinically useful quantifications of the users’ speech production, vocal characteristics, and language. We analyze picture description responses from healthy controls (HC) and patients with two subtypes of MCI: amnestic MCI (aMCI) and non-amnestic MCI (naMCI). Acoustic features were extracted from recordings of picture description responses and language features were extracted from transcripts of the recordings. Classifiers were built from the picture description features to distinguish HC versus MCI or subtypes of MCI. Classification of HC versus aMCI had better performance than HC versus naMCI. Findings suggest that performance of picture description as a screening tool for MCI detection will vary by MCI subtype or by the proportions of various subtypes in an undifferentiated MCI population. We suggest that it is important for future studies to identify and report the subtypes of the MCI cases under analysis. Separate analyses for each MCI subtype would be most useful.

## Introduction

Patients with MCI are a heterogeneous group with different domains and degrees of functional impairment, different etiologies, and different prognoses [1]. An approach to define and characterize subgroups of MCI is to distinguish amnestic MCI (aMCI) where deficits are specifically in memory or non-amnestic MCI (naMCI) where functional deficits are not in the memory domain [1,2].

We suggest that the studies with undifferentiated MCI are hard to interpret because different mixtures of MCI subtypes will have different average functional profiles and thus different associations with individual acoustic or language features. Combinations of features used to distinguish HC from MCI would be tuned to the mixture of subtypes of undifferentiated MCI in a particular study and would not generalize to other populations with different proportions of subtypes. In this study we distinguish aMCI and naMCI cases and analyze these case groups separately. This maintains focus on the study participants’ disease subtypes, with their implications for prognoses and patient care plans.

In this study we use the Miro Health neurobehavioral assessment platform [3] to record spoken responses to an updated version [4] of the “Cookie Theft” picture from the Boston Diagnostic Aphasia Examination (BDAE) [5]. We report the performance of individual features extracted from recorded spoken responses and their transcripts and classifiers built by combining the features at distinguishing healthy controls (HC) from study participants aMCI or naMCI.

Voice acoustics and language usage can provide insight into individuals’ health and cognitive functioning [6,7]. Alzheimer’s disease and related disorders, Parkinson’s spectrum disorders, and MCI can have measurable impacts on voice and language features including those based on voice acoustic qualities, speech production, or lexical and semantic complexity [6]. Alterations in such voice and language features captured in picture descriptions have been associated with diverse populations including normal aging [8], dementia [9,10], primary progressive aphasia (PPA) [4,11], and stroke [4,12]. Characteristic features that distinguish these groups have been identified and machine learning methods have been used to select and combine features to make classifiers that distinguish the groups [13–22].

Collecting spoken language samples from picture description tasks has the benefits of focusing attention, helping to avoid the conflation of problems of speech and language production with memory issues, and allowing analysis of relevant content units such as nouns and verbs specific to scenarios depicted in the pictures as well as general linguistic and acoustic features [10]. Counts of these relevant content units in responses can be especially informative. For instance, patients with dementia of the Alzheimer’s type (DAT) produce fewer of these relevant content units than age-matched controls [23].

A picture description assessment such as Cookie Theft from the Boston Diagnostic Aphasia Examination [5] only requires minutes to administer but requires an administrator with access to and familiarity with scoring rubrics to interpret the test [24,25]. This prevents the wider application of these assessments. An implementation of the picture description task is included as a component of Miro Health’s mobile assessment platform [3,4,26]. This allows remote, self-administration of picture description as well as a battery of additional assessments. Users’ recorded responses are encrypted and sent to Miro Health’s secure servers, where automated analyses of the responses are conducted. Miro Health users’ results are then made available on Miro Health’s secure platform. The results can also be sent to a licensed clinician who ordered the assessment. More details about data security are in Section J in S1 File.

Here we assess the performance of features derived from recordings and transcripts of study participants’ spoken responses to the picture description task at distinguishing aMCI or naMCI from healthy controls. In three separate analyses, cross-validated elastic-net logistic regression [27] is used to select a subset of numerical features and then form a weighted combination of the selected features that optimally separate the HC group from either aMCI, naMCI, or combined MCI (aMCI+naMCI) cases. The performance of these “PD-MCI risk scores” is compared to risk scores formed by a similar machine learning approach but using as input features the scores from a full battery of self-administered neurobehavioral assessments [26], including picture description.

### Related work

There have been other studies that take responses from picture description or related language tasks from study participants, extract numerical features from the responses, and feed the features into statistical or machine learning models to distinguish MCI participants from healthy controls or to make related clinical distinctions. We summarize 12 studies similar to the present work in Table A in S1 File.

Most of the studies shown in Table A in S1 File analyze data collected through administration of picture description tasks, including recordings of participants’ spoken responses, transcripts from the recordings, or written responses. Some studies combine data from picture description tasks with other data including participants’ ages, genders, and responses to other tasks. Several studies use voice or language data but do not use a picture description task: two studies [13,17] work with spontaneous speech, one uses recordings of participants reading a short text [21], and one uses recordings of immediate and delayed retellings of a three-sentence story [20].

An advantage of recording participants’ responses to the picture description task, is that by asking participants for a description of the Cookie Theft scenario or other such circumscribed topics allows predefinition of relevant, informative content units such as objects, actions, and locations depicted in the Cookie Theft picture. By counting participants’ mentions of these relevant content units, semantic information in the participants’ responses is easily coded. These semantic features complement acoustic, lexical, and other language features that would be available from picture description responses or spontaneous speech. These semantic features have been shown to be informative for study participants’ diagnoses, with the ratio of content units to utterances different in MCI and HC groups [28], and the number of content units spoken lower in AD than in HC or MCI [29].

A recording of a spoken language sample could be analyzed directly to extract acoustic features or transcribed and then further processed. Features that can be extracted from transcripts include counts of content units or semantic features, and other language features including counts of words in different lexical categories (noun, preposition, and so on), and measures of sentence length and complexity. Five of the studies in Table A in S1 File collected acoustic and language features and used them in various combinations to separate MCI and HC study participants [14,16–18,20].

In Calza et al, 2021, acoustic, rhythmical, morpho-syntactic and lexical features were calculated from three language tasks. Features were selected for use in a support vector machine (SVM) classifier if they had significantly different distributions in HC and MCI study participants. The performance of the resulting SVM for classification of HC and MCI was (F1 0.74).

In Gosztolya et al, 2019, SVM classifier performance distinguishing HC and MCI using demographic features alone was (F1 0.565). Adding acoustic features improved performance to (F1 0.756) while adding language features alone resulted in a performance of (F1 0.783). Adding both acoustic and language features to the SVM yielded a performance of (F1 0.857).

In Hernández-Domínguez et al, 2018, picture description responses were analyzed to extract phonetic features, vocabulary distributions, counts of words by lexical category, and “information coverage” measures of the relevance of responses to the task. Many models were evaluated but the model with the best average performance for classification of HC and combined cases (MCI or AD) was a support vector machine using linguistic and information coverage features with (AUROC 0.76).

In Roark et al, 2011, performances in classifying HC and MCI participants with SVMs were stronger using “language features” (AUROC 0.731) than input rather than “speech features” (their terms) (AUROC 0.703), but the best classifiers used bother types of features as well as age, education, and 9 additional neuropsychological assessments (AUROC 0.861).

In Frazer et al, 2019, performance on classification of HC and MCI study participants using an SVM built using only language features from transcripts of a picture description response performed slightly better than a SVM built using both acoustic and language features (AUROC 0.73 versus 0.71), but the best performing classifier (AUROC 0.88) used all available features from picture description as well as features extracted from two reading tasks.

The studies mentioned above demonstrate the performance of classifiers built by combining features derived from speech [13,14,16–18] or written language samples [15] to distinguish groups of study participants. If the objective is to identify or distinguish individuals with different medical diagnoses, then the design of classifiers and assessment of classification performance with metrics including AUROCs are paramount. Another study approach is to present statistical tests of the differences in levels of individual features across groups [22,28,29]. The statistical analyses of individual features may give insight into the specific functional deficits in MCI cases. In the present study we assess both the classification performance of speech and language features, or classifiers built from them, and the statistical significance of differences of feature distributions in HC and aMCI or naMCI cohorts.

In addition to the analyses of associations of individual acoustic or language features with diagnosis and to the joint use of many features in machine learning approaches to classify study participants by diagnosis, two of the studies shown in Table A in S1 File, consider joint analysis of pairs of acoustic or language features. In an acoustic analysis [22], the difference between the first harmonic and the third amplitude was different in HC and MCI study participants. In an analysis of transcripts of picture description Cookie Theft responses, the ratio of content words to utterances was different in HC and MCI [28]. In the present study we systematically consider pairs of acoustic or language features. For each contrast, or difference of a pair of normalized features, we evaluate the classification performance and statistical evidence of the contrast across HC, aMCI, and naMCI cohorts.

Of the studies summarized in Table A in S1 File, Calza et al 2021 classifies the MCI participants as either aMCI or multi-domain MCI (mdMCI). Of 32 study participants identified as MCI cases, they distinguish 16 aMCI and 16 multi-domain MCI (mdMCI). However, in their analyses, the MCI subtypes are combined into a single MCI group and contrasted with HC. The other studies either compare HC to undifferentiated MCI or compare HC to combined MCI+AD [18]. We find the studies with undifferentiated MCI are hard to interpret. Combinations of features used by a SVM or other classifier to distinguish HC from MCI would be tuned to the mixture of subtypes of undifferentiated MCI in a particular study and would not generalize to other populations.

In Table A in S1 File we summarize the studies above that are similar to the present work. These studies extract acoustic or language features from study participants’ responses to picture description tasks or related tasks with spoken or written responses, then use these features to distinguish case and control groups among the study participants, or test for differences in values in particular features across case or control groups. Eyigoz et al, 2020 studies incident AD, but the other studies all contrast HC and MCI groups. Hernández-Domínguez et al, 2018 compare a combined (MCI or AD) group to HC, and Jin et al, 2016 Gosztolya et al, 2019 compare both MCI vs HC and AD vs HC. Of these related studies, only Calza et al, 2021 considers different subtypes of MCI.

In the present study we consider both acoustic features extracted from picture description recordings, and language features extracted from transcripts of the recordings. The language features include semantic features based on informative content units, response length, and word counts by lexical category. These features are analyzed individually for association with diagnosis (HC, aMCI, or naMCI) and for ability to classify study participants to their correct diagnosis. We systematically consider the association of diagnoses with contrasts derived from pairs of features. Finally, we select and combine acoustic and language features in optimized classifiers to distinguish HC from aMCI or naMCI.

### The Miro Health assessment platform

The Miro Health platform is a system for remote delivery of neurocognitive assessments, test scoring and analysis, and administrative support for patient management in healthcare or study management in clinical research.

Miro Health assessment batteries may be tailored from a library of self-report questionnaires and more than 40 interactive modules. Miro Health modules include redesigned analogs of legacy neuropsychological tests [26] that have been updated for administration on computer tablets or phones (iOS or Android), and to capture high-fidelity data like movement, speech, language, and response timing information. Each Miro Health module is automatically administered and scored.

## Methods

### Human subject data

Study participants were recruited from neurology clinics at Johns Hopkins University Hospital in Baltimore, Maryland; from participant pools at contract research organization sites; and from the general public through advertisements in newspapers. Screening measures included demographics, medical history (self-reported), the Telephone Interview for Cognitive Status [30,31], the Geriatric Depression Scale [32], the Mayo-Portland Modified Inventory [33,34], and the Mini-Mental State Examination [35,36].

Inclusion criteria for the healthy controls (HC) were age ≥64 years, score of ≥33 on the Telephone Interview for Cognitive Status, English speaker before age 5 years, and high school or equivalent education. Inclusion criteria for the individuals with MCI were age ≥64 years; score of 20–26 on the Mini-Mental State Examination; or medical records with a history of diagnosis of MCI, neurodegenerative disorder, or vascular disorder with cognitive impairment. The individuals with MCI had to meet the American Academy of Neurology [36] clinical criteria for MCI diagnosis. Exclusion criteria for all study participants were evidence of a comorbid neurologic disease; use of drugs known to affect cognition; uncorrected vision or hearing impairment; and history of cancer, substance abuse, or axis 1 disorder.

The study protocol was approved by the Johns Hopkins University Institutional Review Board (protocol 00088299) and the New England Institutional Review Board (protocols 120180208, 120180211, 120180209, and 12080253) and was performed according to the ethical guidelines of the Declaration of Helsinki and its later amendments. All individuals provided informed written consent before enrolling in the study.

### Verification of study participants’ cognitive statuses

Enrolled participants were assigned to a cohort (HC, aMCI, naMCI) using interpretation of neuropsychological test results by an independent, licensed clinical neuropsychologist [1,2,37]. The neuropsychologist had access to eligibility and cohort-assignment brief screen scores—TICS [30,31], MMSE [35,36], or MoCA [38]—demographics, medical history, current medication use, and the results of the full battery of assessments in the Miro Health platform [26]. The numbers of observations by diagnosis, along with age and gender distributions are shown in Table 1. Also shown are the means and standard deviations of three measures of cognitive or functional ability for each diagnosis: MMSE, and scores from verbal learning and memory (VLM) and delayed verbal learning and memory (DVLM) tasks.

### Data collection and preparation

An implementation of the Picture Description task [5,10] is included as the Speak the Scene module in the Miro Health mobile neurobehavioral assessment platform [3,4,26]. An updated picture [4] for the Cookie Theft scenario was used for all study participants.

All participants in this study were assessed with supervision by research assistants at Johns Hopkins University or contract research organizations. Assessments were conducted using Apple iPads running the Miro Health assessment tool and provided by the research site.

On starting the Speak the Scene module, a welcome page with a button labeled “Let’s go” is displayed. On pressing the button, an instructions page is displayed with printed instructions an another “Let’s go” button. A recorded voice reads the instructions out loud when this page is displayed. When study participants press the “Let’s go” button on the touch screen, the Cookie Theft picture is displayed, and the Miro Health application begins recording 90 seconds of the participants’ spoken responses to the stimulus using the iPads’ microphones.

The recorded audio files are automatically encrypted and transmitted to Miro Health’s secure servers for scoring, along with information for study participants’ study registration, eligibility screening, consent, and data from test modules besides picture description. Acoustic analyses proceed automatically. Transcripts are prepared and verified by trained transcriptionists. The users’ transcripts are then automatically processed to extract language features and to score relevant content units related to the picture presented during test administration.

Language features were extracted from the transcripts using Stanford CoreNLP 3.9.1 [39] with Python [40]. Stanford CoreNLP 3.9.1 uses the english-left3words-distsim.tagger. On the CoreNLP website (https://aclweb.org/aclwiki/index.php?title=POS_Tagging_(State_of_the_art))), they note that on the standard WSJ20-24 test set the POS tagger achieves accuracy of 96.97 percent. Feature generation included lexical analysis and scoring of language features specific to the Cookie Theft stimulus such as counts of predefined content units related to the picture. Acoustic analysis of audio files was done using PRAAT 6.1 [41] for Linux. A full list of features that were collected for this study and their definitions is shown in Table L in S1 File. The acoustic and language features were quantile normalized before further analyses.

Several acoustic features that quantify vocal tremor (ATrP, FTrP, ATrI,FTrI, ATrF, FTrF) were not calculated by PRAAT for one or more study participants. Missing values of these features were imputed using the SoftImpute algorithm, separately for each train-test split during optimization of the PD risk scores. [42,43]. Imputed values of features were not used in the evaluations of individual features or in the analysis of normalized differences of features (contrasts).

A total of 44 features to characterize speech acoustics and 47 language features determined from transcripts of study participants’ spoken responses were considered in the following analyses.

### Differences in acoustic and language features across diagnoses

The distributions of values of the features across the three study cohorts were explored graphically by generating violin plots using R and the vioplot library [44].

The features were evaluated for differences in the HC and aMCI or naMCI cohorts. The statistical significance in the difference of each feature’s distributions across diagnoses was tested using logistic regression with adjustment for age and gender in R v. 4.0.2 [45]: glm(y ~ age + gender + feature, family = binomial(link =“logit”)), with y in {case:1, control:0}. The p-values for the regression coefficients associated with the picture description features were used to calculate false discovery rates (FDR) by the Benjamini-Yekutieli procedure [46] as implemented in the p.adjust function in R.

### Classification by diagnosis using individual acoustic and language features

The performance of each picture description feature as a classifier to distinguish the aMCI or naMCI case group from HC was evaluated. For each Miro Health feature, an L2- penalized logistic regression model was trained to predict case or control status using R and the elasticnet library [44] with the Miro Health feature, age, and gender as input variables. The L2 penalty was chosen to minimize cross-validated model deviance: cv.glmnet(x, y, alpha = 0, family =“binomial”, nfolds = 5), with y taking values 1 for MCI cases (either aMCI, naMCI in two separate analyses) and 0 for HC, and x a matrix with columns for the PD feature, age, and gender. Out-of-sample predictions from 5-fold cross-validation with models trained using the selected penalty were generated for each study participant. The procedure was repeated ten times and the results were averaged to give final predictions. These predictions, on the scale of the log-odds of being in an MCI cohort rather than HC, given the input variables, are treated as risk scores for being for MCI, and were evaluated for their classification performance by calculating the area under a receiver operator characteristic curve (AUROC). Classification performance of a test score using measures other than AUROC generally require specification of a threshold on values of the test score such that individuals with scores below the threshold are classified to one group and those with scores above the threshold are classified to another group. We picked thresholds to maximize F1 (harmonic mean of sensitivity and precision) such that specificity is at least 0.85. Using these thresholds, we calculated sensitivity, specificity, precision, accuracy, and F1.

### Contrasts: Pairwise analyses of acoustic and language features from picture description

Differences between pairs of quantile normalized features were calculated and evaluated for association with case or control cohorts by logistic regression, as were the individual features above. These contrasts or differences A-B between two variables A and B distinguish study participants who have high values for variable A relative to their values for variable B or vice versa. Contrasts were calculated for all 47-choose-2 = 1081 pairs of language features based on transcripts and all 44-choose-2 = 946 pairs of speech acoustic features, but not mixed pairs of language and acoustic features. Analyses proceed as for the individual features above. In this study, contrasts of quantile normalized scores were calculated rather than ratios of scores to avoid problems with comparing scores with very different scales or ranges of values and with division by zero or values near zero; and because quantile normalized scores were prepared for input to the elastic net algorithm for generating a combined classifier.

Violin plots were generated to visualize the distributions of contrasts across the three study cohorts using R and the vioplot library.

The statistical significance of the differences in values of the contrasts between cohorts was evaluated with p-values from logistic regression models with adjustment for age and gender. False discovery rates for each contrast were calculated by the Benjamini-Yekutieli procedure. The classification performances of the contrasts for distinguishing HC from the aMCI and naMCI groups were evaluated by calculating AUROCs and other performance measures using 5-fold cross-validated predictions from L2-penalized logistic regression models with the contrast, age, and gender as input variables.

### PD-risk scores: Classifiers built from multiple features extracted from picture description responses

Elastic net logistic regression as implemented in the R library glmnet [27] was used to define three classifiers or risk scores that use acoustics and language features from picture description responses to distinguish HC versus aMCI, HC versus naMCI, and HC versus combined (aMCI + naMCI).

The 47 language features extracted from study participants’ transcripts and 44 acoustic features, as well as age and gender, were used as inputs to train these classifiers. Features were quantile normalized before use. The elastic net logistic regression has an L1- penalty that tends to remove features that do not improve predictive performance, and an L2- penalty that provides further regularization to improve predictive performance. Age and gender were included in the models without penalties, so they were guaranteed to be included in the final risk score models. The form of the final risk score models are a set of weights for a subset of the input features. The risk score calculated as the corresponding weighted sum of input features is an estimate of the log-odds of being a case rather than a control. The parameters alpha (the ratio of L1 to L2 penalties) and lambda (the scaling factor for the penalties) were optimized for classification performance as measured by minimizing predictive deviance with 5-fold cross-validation. In each train-test split of the cross-validation, the training and test subsets had missing values imputed separately. Features with missing values were limited to six acoustic features that quantify vocal tremor (ATrP, FTrP, ATrI,FTrI, ATrF, FTrF). After selecting the optimal elastic net penalties, out-of-sample predictions were made for 10 replications of 5-fold cross-validation. The mean of the 10 model predictions for each study participant was used as their risk score for calculation of AUROC.

Because of low counts of study participants in the two MCI subtypes, 18 aMCI and 15 naMCI, but a larger count of 62 for HC, our primary analyses and development of classifiers or risk scores were for comparisons of HC vs each of the MCI subtypes individually, or HC vs the combined MCI–rather than direct comparisons of aMCI versus naMCI or development of risk scores to distinguish those groups. However, as an alternative approach for developing risk scores from that using penalized logistic regression as described above, we trained and evaluated penalized multinomial logistic regression models for 3-class classification and to simultaneously optimize risk scores for separation of HC versus aMCI, HC versus naMCI, and aMCI versus naMCI. As with the penalized logistic regression models above, the penalized multinomial regression model used was that implemented in the R library glmnet [27]. All 91 acoustic and language features as well as age and gender were used as input features, and three categories of outcomes (HC, aMCI, naMCI) were modeled. L1 and L2 penalties were chosen to minimize predictive deviance with 5-fold cross-validation. Missing values for acoustic measures of vocal tremor were imputed separately for each train-test split during the cross-validation. With the optimized penalties set, model predictions from another 10 rounds of 5-fold cross-validation were averaged to give the liabilities of each study participant for the three diagnoses. Differences of the liabilities for a pair of diagnoses, say liability of aMCI—liability of HC, are estimates of the log-odds of being in one group rather than the other, log-odds of aMCI vs HC in the example. The three differences of liabilities (HC—aMCI, HC—naMCI, and aMCI—naMCI) were used as risk scores for the corresponding classification tasks, and their performances were assessed with AUROCs.

## Results

### Study participants

A total of 95 study participants were assessed on the picture description task for this study. These were distributed across three study cohorts: amnestic MCI (aMCI), non-amnestic MCI (naMCI) and healthy controls (HC). Table 1 shows the counts of study participants across study cohorts and gender, along with the average and range of ages in each cohort. Also shown in Table 1 are means and standard deviations for the MMSE as well as scores from a verbal learning and memory (VLM) task and a delayed verbal learning and memory task (DVLM). The VLM and DVML scores are from a word learning task that is included as a module in the Miro Health assessment platform. The VLM and DVLM scores represent numbers of words correctly recalled. These VLM and DVLM scores have been transformed to represent percentiles in a healthy control reference data set [26].

**Table 1:** Counts, demographics, and functional measures of study participants in the aMCI, naMCI, and HC cohorts.

Cohort	Participants	Female / Male	Age Mean (sd)	MMSE Mean (sd)	VLM Largest Correct Set Spoken Mean (sd)	DVLM Largest Correct Set Spoken Mean (sd)
aMCI	18	6 / 12	70.2 (7.4)	25.8 (2.6)	28.7 (23.7)	29.1 (17.5)
naMCI	15	6 / 9	71.5 (5.8)	27.4 (2.5)	41.8 (22.8)	37.0 (23.2)
HC	62	35 / 27	70.3 (4.5)	29.2 (1.1)	58.9 (27.3)	57.9 (28.7)

**HC** = healthy controls. **aMCI** = amnestic mild cognitive impairment. **MCI** = mild cognitive impairment. **naMCI** = non-amnestic mild cognitive impairment. **VLM** = Verbal Learning & Memory. **DVLM** = Delayed Verbal Learning & Memory. **MMSE** = Mini-Mental State Examination.

### Data preparation

The picture description responses were processed to generate 91 quantitative features. Of these features, 44 were acoustic features calculated from the study participants’ recorded responses, and 47 were language features generated from transcripts of the voice recordings, counts of sentences, words, and syllables; lexical analyses with counts of words in different categories or parts of speech; and counts of content units or words for items and actions in the responses. The means and standard deviations of each feature are shown for the three diagnostic groups in Table B in S1 File. The features were quantile normalized before further analysis. The means and standard deviations of the quantile normalized features in the three diagnostic groups are shown in Table C in S1 File.

As seen in Tables 2 and 3 various sets of content units are distinguished and counted separately. If content units are related to the scenario depicted in the picture, they are labeled “RELATED”, while combined counts of related and unrelated content are labeled “ALL”. Features that include “DISTINCT” in the name do not count repeated uses of a content unit. Features that include “RIGHT” or “LEFT” in the name are counts of content units related to items on the corresponding side of the picture, such as “dog” on the left or “sink” on the right for the Cookie Theft scene. Features that include “Both Sides” in the name are counts of content units that are not localized to one side of the picture, such as “kitchen” or “family” for the Cookie Theft scene.

**Table 2:** Association of features derived from picture description recordings or transcripts with diagnosis of aMCI or HC.

Variable	p-value	FDR	AUROC	Effect Direction
Count of DISTINCT RELATED content units	1.1E-04	0.019	0.82	(-)
Count of RELATED content units	1.2E-04	0.019	0.83	(-)
Count of indefinite articles	1.7E-04	0.019	0.82	(-)
Count of prepositions	2.1E-04	0.019	0.79	(-)
Count of nouns	2.3E-04	0.019	0.81	(-)
Both Sides Total Content Units DISTINCT	2.5E-04	0.019	0.80	(-)
Both Sides Total Content Units	3.3E-04	0.021	0.78	(-)
Count of ALL content units	3.7E-04	0.021	0.76	(-)
Count of content units on RIGHT side of picture	4.1E-04	0.021	0.75	(-)
Count of Syllables	6.0E-04	0.028	0.74	(-)
Count of words	7.9E-04	0.032	0.73	(-)
Count of ALL DISTINCT content units	8.2E-04	0.032	0.73	(-)
Count of DISTINCT content units on RIGHT side of picture	1.1E-03	0.039	0.71	(-)
Count of sentences	1.5E-03	0.049	0.69	(-)
Count of phrases	1.6E-03	0.049	0.71	(-)
Acoustics standard deviation of F0	2.8E-03	0.080	0.71	+
Count of complete sentences	4.3E-03	0.113	0.66	(-)
Count of content units on LEFT side of picture	4.4E-03	0.113	0.66	(-)
Count of all function words	4.9E-03	0.113	0.67	(-)
Count of DISTINCT content units on Left side of picture	4.9E-03	0.113	0.65	(-)

**HC** = healthy controls. **aMCI** = amnestic mild cognitive impairment. **MCI** = mild cognitive impairment. **FDR** = Benjamini Yakutieli false discovery rate. **AUROC** = area under the receiver operator characteristic curve.

**Table 3:** Association with membership in the aMCI or HC cohorts of contrasts (A-B) of normalized values of two speech or language features A and B.

Speech or Language Feature A	Speech or Language Feature B	p-value	FDR	AUROC	Effect Direction
Count of modals	Count of prepositions	4.9E-05	0.114	0.86	1
Count of modals	Count of RELATED content units	7.5E-05	0.114	0.89	1
Pro-sentences	Count of prepositions	8.5E-05	0.114	0.84	1
Count of modals	Count of indefinite articles	8.6E-05	0.114	0.86	1
Count of modals	Count of nouns	1.1E-04	0.114	0.88	1
Pro-sentences	Count of RELATED content units	1.1E-04	0.114	0.85	1
Count of modals	Both Sides Total Content Units Distinct	1.1E-04	0.114	0.85	1
Count of modals	Count of words	1.1E-04	0.114	0.84	1
Syllables per word min	Count of DISTINCT RELATED content units	1.1E-04	0.114	0.83	1
Count of modals	Count of content units on RIGHT side of picture	1.2E-04	0.114	0.84	1
Count of modals	Count of Syllables	1.2E-04	0.114	0.84	1
Syllables per word min	Count of RELATED content units	1.2E-04	0.114	0.84	1
Pro-sentences	Count of DISTINCT RELATED content units	1.3E-04	0.114	0.84	1
Pro-sentences	Count of Syllables	1.3E-04	0.114	0.81	1
Count of modals	Count of ALL content units	1.3E-04	0.114	0.84	1
Pro-sentences	Count of nouns	1.3E-04	0.114	0.86	1
Count of modals	Both Sides Total Content Units	1.3E-04	0.114	0.83	1
Count of modals	Count of ALL DISTINCT content units	1.4E-04	0.114	0.83	1
Pro-sentences	Count of ALL content units	1.4E-04	0.114	0.82	1
Count of modals	Count of DISTINCT RELATED content units	1.5E-04	0.114	0.89	1

**HC** = healthy controls. **aMCI** = amnestic mild cognitive impairment. **MCI** = mild cognitive impairment. **FDR** = Benjamini Yakutieli false discovery rate. **AUROC** = area under the receiver operator characteristic curve.

### Analyses of individual acoustic and language features

The acoustic and language features were assessed to determine their association with the study participants’ health statuses. For each feature, the statistical significance of the differences between aMCI and HC cohorts was assessed using logistic regression with adjustments for age and gender. Table 2 shows the 20 features having p-values less than 0.005. Included in this set of significant associations are ten features having “content units” in their names and representing various counts of content units in the study participants’ responses or the “on-topic” relevance of their response. There are four lexical features or counts of words from various parts of speech: indefinite articles, prepositions, nouns, and function words. One score is an acoustic feature—the standard deviation of the fundamental frequency of the participants voice during the recorded response. The remaining features are different measures of the length of the response: syllables, words, phrases, sentences, and complete sentences.

The 20 scores with p-values below 0.005 represent 20 out of the 91 features considered in the analysis. To complement the p-values and account for multiple testing, in Table 2 we provide false discovery rates (FDRs). An FDR represents an estimate of the fraction of features at that FDR’s row or above in Tables 2 or 3 that are false positives. The features and the tests that yielded the p-values of Table 2 are not independent because they are all extracted from the same responses, so the conservative Benjamini-Yekutieli procedure [46] that allows for dependent tests was used. Table D in S1 File is an expanded version of Table 2 that shows p-values and FDRs for all 91 features. Panels A and B in Fig A in S1 File show histograms of the p-values and FDRs for all 91 features.

For each feature, the statistical significance of the differences between naMCI and HC cohorts was also assessed using logistic regression with adjustments for age and gender. As shown in Table E and in panels A and B of Fig B in S1 File, the distribution of p-values for these association tests with the naMCI and HC cohorts showed few nominally significant features, with p-values below 0.05, and zero features with estimated FDRs below 1. This suggests that any findings from this analysis contrasting the naMCI and HC groups would be false positives. There are also fewer participants in the naMCI cohort than in the aMCI cohort. For these reasons, the analyses of features considered here are focused on identifying and characterizing differences between the aMCI and HC cohorts rather than analyses using the naMCI cohort.

Even if there is statistical evidence that an acoustic or language feature is different across aMCI and HC cohorts, it may not be particularly useful for distinguishing the cohorts. The practical significance of each feature for detection of cohort membership was determined by using the feature as a classifier. In any practical classification task using these scores, age and gender should be available for the subject under evaluation, so L2-penalized logistic regression classifiers were made using age, gender, and a single acoustic or language feature as predictors. Classifier performance was quantified with area under the receiver-operator characteristic curve (AUROC) for an average score from 10 iterations of 5-fold cross-validation. For comparison, a penalized logistic regression model using only age and gender to identify cases is used as the baseline. That baseline model for separating the aMCI and HC cohorts has an AUROC of 0.61. The features presented in Table 2 improve the classifier performance over the baseline model to as high as 0.83, but at least 0.65. Note that the degree of statistical significance (low p-value and low FDR) is sorted in almost the same order as the degree of practical significance as a classifier (high AUROC).

The final column in Table 2 is labeled “effect direction” and represents the sign of the estimated regression coefficient for the acoustic or language feature in the logistic regression model that was used to calculate the p-values. For the analyses, aMCI cases were coded as 1 and controls were coded as 0, so a positive effect direction indicates that aMCI cases typically have higher values of the feature than controls, after adjusting for age and gender. All but one of the features shown in Table 2 have negative effect directions, so controls tend to have higher values for these features. The features with negative effect directions in Table 2 are all counts of various quantities in the picture description transcripts, so they could indicate that the controls just have longer responses than the aMCI participants. That suggests that more insight may come from considering ratios or contrasts from pairs of features. The sole feature in Table 2 with a positive effect direction is “Acoustic standard deviation of F0.” This result shows that the participants in the aMCI cohort tended to have more variation in the fundamental frequency or pitch of their voices over the course of their responses. AUROCs and effect directions for analyses of all 91 acoustic and language features for distinguishing aMCI and HC cohorts are shown in Table D in S1 File. The results of corresponding analyses of features for distinguishing naMCI and HC cohorts are shown in Table E in S1 File.

To further characterize the classification performance of each of the 91 acoustic or language features when used along with age and gender to classify aMCI vs HC, additional performance measures were calculated. Accuracy, sensitivity, specificity, precision, and F1 are shown in Table G in S1 File. The threshold on the risk score needed to calculate these other measures was chosen to maximize F1 such that specificity was at least 0.85. Similarly, these performance measures were calculated for classification of naMCI vs HC by each acoustic or language feature, with results shown in Table H in S1 File.

**Fig 1:**
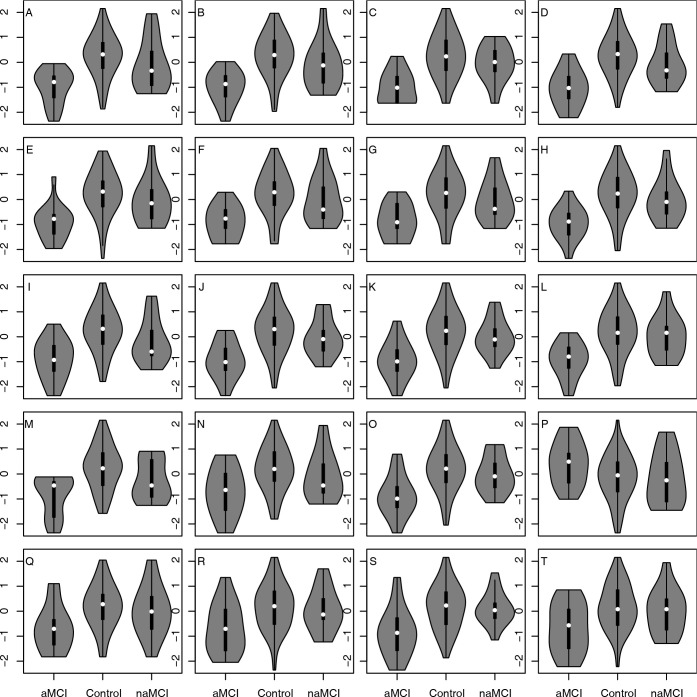
The 2018 speech or language features from Table 2 are displayed with violin and embedded box plots to show the distribution of these features in the three study cohorts: aMCI, naMCI, and Healthy Control. These features have p-values below 0.005 for association with membership in the aMCI or HC cohorts, as determined by logistic regression with adjustment for age and gender. The plots are in the same alphanumeric order as the rows in Table 2, with panel A showing the distribution across cohorts of values for Count of DISTINCT RELATED content units, and subsequent panels for features with increasing p-values. The corresponding p-values for the association of features with aMCI versus HC cohort status are in Table 1. No features had false discovery rates below 1.0 for association with naMCI versus HC cohort status. A: Count of DISTINCT RELATED content units. B: Count of RELATED content units. C: Count of indefinite articles. D: Count of prepositions. E: Count of nouns. F: Both Sides Total Content Units DISTINCT. G: Both Sides Total Content Units. H: Count of ALL content units. I: Count of content units on RIGHT side of picture. J: Count of Syllables. K: Count of words. L: Count of ALL DISTINCT content units. M: Count of DISTINCT content units on RIGHT side of picture. N: Count of sentences. O: Count of phrases. P: Acoustics standard deviation of F0. Q: Count of complete sentences. R: Count of content units on LEFT side of picture. S: Count of all function words. T: Count of DISTINCT content units on Left side of picture.

The distributions across the three cohorts of the features from Table 2 are displayed in Fig 1 using violin plots with embedded box plots. The features are displayed in the same order in both Table 2 and Fig 1, with “Count of DISTINCT RELATED content units” in the first row of Table 1 and in panel A of Fig 1 and so on. In each panel, three violin plots are shown, representing from left to right the aMCI cohort, the HC cohort, and the naMCI cohort. These features were selected for being different across the aMCI and HC cohorts, and visual comparison of the left and middle plots in each panel confirms that there is difference in typical values of these features across those two cohorts. By contrast, the naMCI features that form the right plots in each panel are less dissimilar to the HC scores than the aMCI features.

### Contrasts of paired acoustic or language features

Analyses of contrasts between pairs of features may be complementary to analyses of individual features. For example, two talkative study participants may have both high counts of words and high counts of sentences. But the first participant may have a word count 1 standard deviation above the mean and a sentence count 2 standard deviations above the mean, while the second participant has a word count 2 standard deviations above the mean and a sentence count 1 standard deviation above the mean. All features considered in this study are quantile normalized, so their effective units are standard deviations from the mean. In this scenario, the first participant has a contrast for words—sentences of (1–2 = -1) while the second has a contrast for words—sentences of (2–1 = 1). The different values and signs of the contrasts indicate that the first participant has few words for many sentences, or short sentences, while the second participant has many words for few sentences, or long sentences.

Such contrasts were calculated for all 946 pairs of acoustic features based on the audio recordings and for all 1081 pairs of language features based on the response transcripts. Each contrast was generated as the difference (A-B) between the quantile normalized feature A named in the first column of Table 3 and the feature B named in the second column.

These contrasts were analyzed to assess their ability to distinguish the aMCI and HC cohorts, just as the individual features were assessed to generate the results in Table 2. Results of the regression and classification analyses for the contrasts are shown in Table 3.

**Fig 2:**
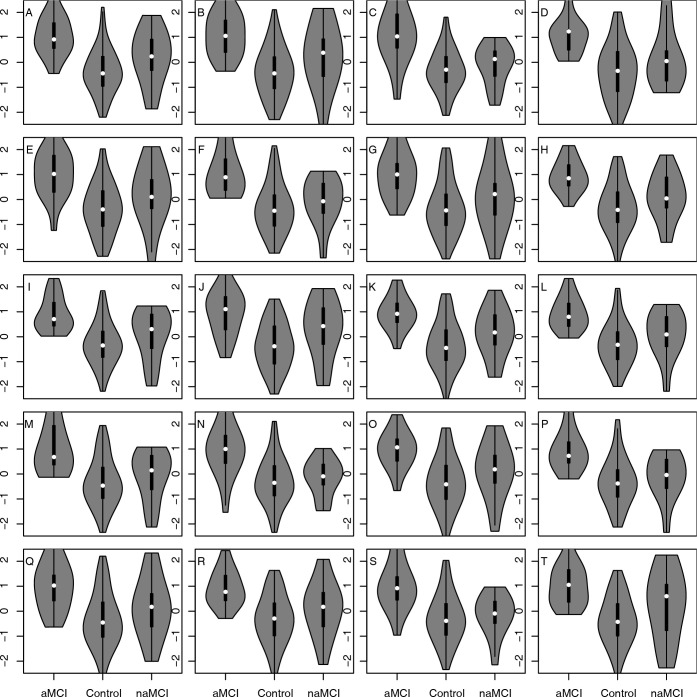
Contrasts of speech or language features from Table 3 with the 20 most significant p-values for association with aMCI or HC cohort membership are displayed with violin and embedded box plots. The corresponding p-values are in Table 3. No contrasts had false discovery rates below 0.5 for association with naMCI versus HC cohort membership. The plots are in the same alphanumeric order as the rows in Table 3, with panel A showing the distribution across cohorts of values for the difference of quantile normalized values of Count of modals minus Count of prepositions, and subsequent panels for contrasts with increasing p-values. A: Count of modals minus Count of prepositions. B: Count of modals minus Count of RELATED content units. C: Pro-sentences minus Count of prepositions. D: Count of modals minus Count of indefinite articles. E: Count of modals minus Count of nouns. F: Pro-sentences minus Count of RELATED content units. G: Count of modals minus Both Sides Total Content Units DISTINCT. H: Count of modals minus Count of words. I: Syllables per word min minus Count of DISTINCT RELATED content units. J: Count of modals minus Count of content units on RIGHT side of picture. K: Count of modals minus Count of Syllables. L: Syllables per word min minus Count of RELATED content units. M: Pro-sentences minus Count of DISTINCT RELATED content units. N: Pro-sentences minus Count of Syllables. O: Count of modals minus Count of ALL content units. P: Pro-sentences minus Count of nouns. Q: Count of modals minus Both Sides Total Content Units. R: Count of modals minus Count of ALL DISTINCT content units. S: Pro-sentences minus Count of ALL content units. T: Count of modals minus Count of DISTINCT RELATED content units.

The contrasts were used as predictor variables in logistic regression models. As before, the outcome for the regression analyses was cohort membership coded as aMCI = 1 and HC = 0, and age and gender were used as adjustment covariates. The p-values for the contrasts from the logistic regression of aMCI versus HC were calculated and used to sort the results of Table 3, with the most significant results at the top of the table. Table 3 shows 20 contrasts with p-values below 1.5e-4. Violin plots showing the distributions of these 20 contrasts are shown in Fig 2. Table F in S1 File shows a longer set of results with 61 contrasts having p-values below 5e-4. To address multiple testing and dependencies between the tests FDRs were calculated to complement the p-values using the Benjamini-Yekutieli procedure. The resulting FDR estimates are included in Table 3. Histograms of p-values and FDRs for all 2007 contrasts evaluated in these analyses are shown in panels C and D of Fig B in S1 File. The practical significance of these contrasts for separating aMCI and HC study participants was assessed by making classifiers of 5-fold cross-validated predictions from L2-penalized logistic regression models with the contrast, age, and gender as input variables. The baseline model with only age and gender as input variables has an AUROC of 0.61, but addition of the contrasts to the model results in AUROCs as high as 0.89 for the contrast of (count of models [modal verbs]—count of related content units).

The effect direction or the sign of the estimated regression coefficient from the logistic regression that yielded the p-values is shown. The outcomes for the logistic regression were coded as aMCI = 1 and HC = 0, so a positive effect direction indicates that members of the aMCI tended to have higher values of the contrast than controls, or that aMCI cases tended to have relatively high values of the features in column A of Table 3, while controls tended to have relatively high values of features in column B.

The corresponding p-values, FDRs and classification AUROCs were calculated for analyses distinguishing each contrast’s association with naMCI versus HC status. As shown in Panels C and D of Fig B in S1 File, zero contrasts have FDRs below 1 for association with naMCI versus HC status.

The violin plots in Figs 1 and 2 show the distribution of features and contrasts respectively, without adjustment for age and gender. The analyses represented in Tables 2 and 3 do account for age and gender, and a reasonable prior is that the adjustment for these factors is important for at least some of the features or contrasts. For example, voice pitch or fundamental frequency varies with age and gender. The acoustic features and contrast with highest significance in the analyses for Tables 2 and 3 were the standard deviation of the fundamental frequency F0, speech breaks, and their contrast. To visually show the effects of gender on these measures, violin plots of standard deviation in F0, speech breaks, and their contrast are shown in Fig C in S1 File, stratified by cohort (aMCI or HC) and gender.

### Classification by diagnosis using weighted combinations of features

The contrasts presented in Table 3 show more significant separation of the aMCI and HC cohorts than do the individual features in Table 2, with lower p-values and higher AUROCs. Extending the idea that some features provide complementary information about cohort membership or are most informative when their relative levels are considered, a linear combination of a larger set of features was constructed, with optimization for selection of the most informative set of features to use and for the weights to put on each feature in the combination.

The elastic net logistic regression algorithm was used to find an optimized weighted combination of speech and language features for separation of the aMCI and HC cohorts. These model predictions are on the scale of log-odds of being in the aMCI cohort versus HC cohort, based on age, gender, and features extracted from picture description data. The algorithm’s tuning parameters or penalties were chosen by 5-fold cross validation. After selection of the penalties, an out-of-sample prediction was made for each study participant in the aMCI and HC cohorts by averaging out-of-sample predictions from another 10 iterations of 5-fold cross-validation of the penalized logistic regression. We name these predictions the “PD aMCI risk scores”. We used the same procedure to make risk scores for distinguishing the HC cohort from naMCI, and for separating HC from combined (aMCI + naMCI), for three PD risk scores.

During cross-validation, missing values in acoustic features measuring voice tremor were imputed separately for each train-test split. Age and gender were not penalized in the elastic net fitting procedure, so they were forced to be in the final prediction model. The full set of 91 picture description features was used as input to the elastic net fitting procedure, but final classification models only used an informative subset of features. The selected features and their weights are shown in Table K in S1 File.

These PD risk scores were used to calculate the AUROCs for separating pairs of study cohorts, as shown in Table 4. For comparison, the AUROCs for separation of these cohorts were also calculated for a second set of risk scores based on input features from a large battery of neurobehavioral tests implemented in the Miro Health assessment platform and described in previous work [24]. As shown in Table 4, the PD risk scores do not have classification performance as strong as the risk score based on a large battery of assessments.

**Table 4:** Performance of MCI risk scores at separating cohorts of study participants.

Classification task	PD risk score AUROC	Miro Health risk score AUROC
aMCI vs HC	0.88	0.97
naMCI vs HC	0.61	0.80
(aMCI+naMCI) vs HC	0.74	0.89

**HC** = healthy controls. **aMCI** = amnestic mild cognitive impairment. n**aMCI** = non-amnestic mild cognitive impairment. **MCI** = mild cognitive impairment. **AUROC** = area under the receiver operator characteristic curve. **PD** = Picture Description. Additional performance metrics for these classifiers are shown in Table J in S1 File.

**PD risk score**: out of sample predictions from models trained to separate MCI (or subtype) and HC. Estimated as the log-odds of MCI:HC given speech and language features from PD, age and gender.

**Miro Health risk score**: out of sample predictions from models trained to separate aMCI and HC [26]. Estimated as the log-odds of MCI:HC given features from PD as well as scores from the other neurobehavioral test modules on the Miro Health assessment platform, age and gender.

An alternative approach was used to make a second set of PD risk scores. In this analysis, penalized multinomial regression was used to simultaneously find combinations of age, gender, and acoustic or language scores from picture description for classifying aMCI vs HC, naMCI vs HC, and aMCI vs naMCI. The performances at separating these three groups as measured by AUROC under 5-fold cross-validation are 0.86, 0.51, and 0.78 respectively, as shown in Table I in S1 File.

## Discussion

Our analyses suggest that performance of automated classifiers built on features extracted from picture description as a screening tool for MCI detection will vary greatly as a function of the proportions of MCI subtypes in a study or clinical source population. We used elastic-net logistic regression [27] to screen and combine 91 numerical features extracted from picture description responses to make classifiers that distinguish MCI cases from healthy controls (HC). Three separate analyses were done to consider different subtypes of MCI: HC versus aMCI, HC versus naMCI and HC versus combined MCI (aMCI+naMCI). The approach was to use predictions from penalized logistic regression models based on age, gender, and the acoustic and language features as measures of each study participant’s risk of being a case. These predictions are estimates of an individual’s log-odds of being a case, with higher values associated with cases and lower values associated with controls.

Evaluating these predictions, here called PD MCI risk scores, as classifiers we find that the PD-aMCI risk score has an AUROC of 0.88 for separating aMCI cases from HC, but the PD-naMCI risk score has an AUROC of only 0.61 for separating naMCI cases from HC. The observed AUROC for separation of combined aMCI and naMCI, with 18 and 15 cases respectively, from HC is 0.74. This AUROC for distinguishing HC from MCI cases with mixed subtypes is intermediate between the performance of PD risk scores at classifying HC vs aMCI (stronger at 0.88) and HC vs naMCI (weaker at 0.61). Tables D and E and Figs A and B in S1 File show that more of the features extracted from picture description responses had high AUROCs or low p-values for classification of study participants by diagnosis in the HC vs aMCI analyses than in the HC vs naMCI analyses.

These results suggest that distinguishing aMCI from HC is an easier problem than distinguishing naMCI from HC, when using picture description responses to make the distinctions. This is in agreement with analyses using response to other neuropsycological tests to distinguish HC from either aMCI or naMCI, where classification performance was stronger for HC vs aMCI [24]. This suggests that the reported performance of methods to detect MCI or distinguish MCI from HC will depend on the proportions of aMCI and naMCI subtypes in a set of study participants with MCI. We propose that studies of MCI detection should assess and report counts of MCI cases by subtype.

Our results using classifiers based on acoustic and language features are compatible with results from prior studies as summarized in Table A in S1 File. Looking at studies that report AUROCs for classification of HC versus MCI, Asgari et al [13] report an AUROC of 0.796, Fraser et al [16] report 0.73, and Roark et al [20] report 0.703. These results are similar to the AUROC of 0.74 for classification of HC vs combined MCI (aMCI+naMCI) in the present study. The other studies in Table A in S1 File did not determine the MCI subtypes of their study participants and analyze the subtypes separately. Calza et al [14] did distinguish aMCI (memory impaired, other cognitive domains not impaired) cases from multiple-domain MCI (two or more domains of cognitive function impaired, possibly including memory), but separate analyses were not done for the two identified subtypes.

Analyses of individual acoustic or language features show ability to distinguish aMCI from HC, but not to distinguish naMCI from HC. Many scores extracted from picture description responses are significantly associated with aMCI versus HC cohort, as demonstrated with low p-values and FDRs for logistic regression analyses in Table 2. Many contrasts of scaled scores are also associated with aMCI versus HC cohort as shown in Table 3. None of these scores or contrasts are significantly associated with naMCI versus HC cohort; all of them have estimated FDRs of 1 in the logistic regression analyses suggesting that any findings would be false positives. Given the small number of naMCI cases, 15, in this study we cannot conclude that there are no acoustic or language features that could distinguish naMCI from HC, but this study was not able to find any.

In this study, analyses suggest that language features based on picture description transcripts may be more informative for distinguishing MCI cases from controls than acoustic features from the voice recordings. In the logistic regression analyses of association of individual features with aMCI vs HC status by logistic regression, the only acoustic feature among the top twenty features with the most significant p-values as shown in Table 2 is “Acoustics standard deviation of F0”. Tables D in S1 File show that acoustic features (with “acoustics” in their feature names) have higher p-values from the association tests with aMCI vs HC status and lower AUROCs when used as classifiers than many of the non-acoustic features. However, the risk scores for distinguishing aMCI vs HC and combined MCI (aMCI + naMCI) vs HC include both acoustic and language features. Table K in S1 File shows the variables used in the risk score models and their weights. Together the results suggest that the most informative individual features for distinguishing aMCI from HC are mainly language features extracted from transcripts, but that there is complementary information for separating aMCI from HC in the acoustic features.

The AUROCs in Table E in S1 File show that no individual features were particularly good at distinguishing naMCI from HC. As shown in Table K in S1 File, the risk score for distinguishing naMCI from HC selected by 5-fold cross-validation is just the baseline model with age and gender as predictors but no picture description features. As naMCI cases include those with language deficits (as opposed to memory deficits in the definition of aMCI status), we expect language and acoustic features to characterize at least some naMCI cases, but we will have to revisit this hypothesis with a larger sample of naMCI cases.

We see pronounced differences in the ability to distinguish MCI cases from HC depending on the subtypes of the cases. We suggest that it is important for future studies to identify and report the subtypes of the MCI cases under analysis. Separate analyses for each MCI subtype, and analyses to distinguish the subtypes would be most useful. Reporting the distribution of subtypes among the MCI cases as in Calza et al, 2021 would at least allow readers to evaluate the relevance of findings to their own clinical or research efforts.

With only 18 aMCI and 15 naMCI participants in the study, we did not focus on differentiating MCI subtypes in our analysis. However, the alternative construction we used for generating risk scores–a penalized multinomial model trained with all three outcomes–gives risk scores for distinguishing aMCI versus HC, naMCI versus HC, and aMCI versus naMCI. As shown in Table I in S1 File, the AUROC for separating aMCI versus naMCI using the multinomial risk score is 0.78.

The large number of features that can be extracted from picture description responses allow rich narrative descriptions of the functional deficits an individual is experiencing with speech or language. These scores span several dimensions of study participants’ responses to the picture description task: counts of informative content units relevant to the Cookie Theft picture (e.g. “Count of DISTINCT RELATED content units”), voice stability (e.g. “Acoustics standard deviation of F0”), and rate of speech (e.g. “Count of words”). Contrasts or differences between normalized versions of individual Miro Health features give additional insight into the study participants’ performance.

We know that language samples can provide us with detailed information about a patient’s expressive language. We expect that the ease of remote self-administration of the Miro Health implementation of the picture description task, with uniform test scoring for language feature extraction, and collection of acoustic features will allow the expanded and routinized use of acoustic and language evaluation in research and in the clinic.

### Limitations and future work

Classification results based on the features from picture description and the combined PD risk scores may be different in application or studies of Miro Health users with different distributions of demographic and health covariates or with more Miro Health users in the borderline region between healthy and MCI status.

Here we focus on distinguishing healthy controls from study participants with amnestic mild cognitive impairment (aMCI) and non-amnestic mild cognitive impairment (naMCI). Only 15 study participants were in the naMCI cohort and there were no study participants with other conditions or diagnoses. In future work we will evaluate additional patient groups likely to have different spectra of effects across the collection of features than were seen in the healthy control and aMCI cohorts—larger numbers of naMCI study participants, and several groups with distinct forms of aphasia and apraxia of speech.

The individual acoustic and language features and the differences in pairs of features whose classification performances are summarized in Tables G and H respectively in S1 File were pre-specified so only the covariate adjustment (age and gender) was subject to over-fitting (that is, there was no variable selection). Thresholds for calculation of classification performance measures were set to maximize F1 with the constraint that specificity is at least 0.85. There is a trade-off between specificity, sensitivity and precision; as a potential screening tool, we constrained the analyses to have high specificities. With the constraint for high specificity, the other measures sensitivity, precision, and F1 can be low.

There are many types of classifiers that could be used to predict diagnosis given a list of acoustic and language features as input, such as support vector machines, random forests, and neural networks. We intend to explore these and other models when we have more data available. Given the small data set in hand, we focused on combining scores to make classifiers for separating HC from the MCI case groups that are easily interpretable. The alternative construction of risk scores used here, penalized multinomial regression with 3 diagnoses (HC, aMCI, naMCI) allows multi-class classification and pairwise aMCI vs naMCI classification (AUROC 0.78), but proper distinction of these subtypes requires specific consideration of the domains of cognitive impairment: memory for aMCI, domains other than memory for naMCI, and multiple domains for mdMCI.

## Supporting information

S1 File (DOCX)

## References

[1] Roberts R, Knopman DS. Classification and epidemiology of MCI. Clin Geriatr Med. 2013 Nov;29(4):753–72. doi: 10.1016/j.cger.2013.07.003 24094295 PMC3821397

[2] Jak AJ, Preis SR, Beiser AS, Seshadri S, Wolf PA, Bondi MW, Au R. Neuropsychological Criteria for Mild Cognitive Impairment and Dementia Risk in the Framingham Heart Study. J Int Neuropsychol Soc. 2016 Oct;22(9):937–943. doi: 10.1017/S1355617716000199 27029348 PMC5045758

[3] Miro Health [Internet]. www.mirohealth.com. [cited 2022 Apr 6]. Available from: https://www.mirohealth.com/

[4] Berube S, Nonnemacher J, Demsky C, Glenn S, Saxena S, Wright A, et al. Stealing Cookies in the Twenty-First Century: Measures of Spoken Narrative in Healthy Versus Speakers With Aphasia. American Journal of Speech-Language Pathology [Internet]. 2019 Mar 11 [cited 2021 Apr 18];28(1S):321–9. Available from: https://pubmed.ncbi.nlm.nih.gov/30242341/ doi: 10.1044/2018_AJSLP-17-0131 30242341 PMC6437702

[5] Goodglass H, Kaplan E, Weintraub S. BDAE: The Boston Diagnostic Aphasia Examination.3rd ed. Philadelphia, PA: Lippincott Williams & Wilkins; 2001

[6] Voleti R, Liss JM, Berisha V. A Review of Automated Speech and Language Features for Assessment of Cognitive and Thought Disorders. IEEE Journal of Selected Topics in Signal Processing [Internet]. 2020 Feb 1 [cited 2022 Mar 21];14(2):282–98. Available from: https://arxiv.org/abs/1906.01157 doi: 10.1109/jstsp.2019.2952087 33907590 PMC8074691

[7] Martínez-Nicolás I, Llorente TE, Martínez-Sánchez F, Meilán JJG. Ten Years of Research on Automatic Voice and Speech Analysis of People With Alzheimer’s Disease and Mild Cognitive Impairment: A Systematic Review Article. Frontiers in Psychology [Internet]. 2021 Mar 23;12. Available from: https://www.ncbi.nlm.nih.gov/pmc/articles/PMC8021952/ doi: 10.3389/fpsyg.2021.620251 33833713 PMC8021952

[8] Cooper PV. Discourse Production and Normal Aging: Performance on Oral Picture Description Tasks. Journal of Gerontology. 1990 Sep 1;45(5):P210–4. doi: 10.1093/geronj/45.5.p210 2394918

[9] Forbes-McKay KE, Venneri A. Detecting subtle spontaneous language decline in early Alzheimer’s disease with a picture description task. Neurol Sci [Internet]. 2005;26(4):243–54. Available from: 10.1007/s10072-005-0467-9 16193251

[10] Giles E, Patterson K, Hodges JR. Performance on the Boston Cookie theft picture description task in patients with early dementia of the Alzheimer’s type: Missing information. Aphasiology. 1996 May;10(4):395–408.

[11] Weintraub S. Primary Progressive Aphasia. Archives of Neurology. 1990 Dec 1;47(12):1329. 2252450 10.1001/archneur.1990.00530120075013

[12] Yorkston KM, Beukelman DR. An Analysis of Connected Speech Samples of Aphasic and Normal Speakers. Journal of Speech and Hearing Disorders. 1980 Feb;45(1):27–36. doi: 10.1044/jshd.4501.27 7354627

[13] Asgari M, Kaye J, Dodge H. Predicting mild cognitive impairment from spontaneous spoken utterances. Alzheimer’s & Dementia: Translational Research & Clinical Interventions. 2017 Jun;3(2):219–28. doi: 10.1016/j.trci.2017.01.006 29067328 PMC5651423

[14] Calzà L, Gagliardi G, Rossini Favretti R, Tamburini F. Linguistic features and automatic classifiers for identifying mild cognitive impairment and dementia. Computer Speech & Language. 2021 Jan;65:101113.

[15] Eyigoz E, Mathur S, Santamaria M, Cecchi G, Naylor M. Linguistic markers predict onset of Alzheimer’s disease. EClinicalMedicine. 2020 Oct;100583. doi: 10.1016/j.eclinm.2020.100583 33294808 PMC7700896

[16] Fraser KC, Lundholm Fors K, Eckerström M, Öhman F, Kokkinakis D. Predicting MCI Status from Multimodal Language Data Using Cascaded Classifiers. Frontiers in Aging Neuroscience. 2019 Aug 2;11. doi: 10.3389/fnagi.2019.00205 31427959 PMC6688130

[17] Gosztolya G, Vincze V, Tóth L, Pákáski M, Kálmán J, Hoffmann I. Identifying Mild Cognitive Impairment and mild Alzheimer’s disease based on spontaneous speech using ASR and linguistic features. Computer Speech & Language. 2019 Jan;53:181–97.

[18] Hernández-Domínguez L, Ratté S, Sierra-Martínez G, Roche-Bergua A. Computer-based evaluation of Alzheimer’s disease and mild cognitive impairment patients during a picture description task. Alzheimer’s & Dementia: Diagnosis, Assessment & Disease Monitoring. 2018;10:260–8. doi: 10.1016/j.dadm.2018.02.004 29780871 PMC5956933

[19] König A, Satt A, Sorin A, Hoory R, Toledo-Ronen O, Derreumaux A, et al. Automatic speech analysis for the assessment of patients with predementia and Alzheimer’s disease. Alzheimer’s & Dementia: Diagnosis, Assessment & Disease Monitoring. 2015 Mar 1;1(1):112–24. doi: 10.1016/j.dadm.2014.11.012 27239498 PMC4876915

[20] Roark B, Mitchell M, Hosom JP, Hollingshead K, Kaye J. Spoken language derived measures for detecting mild cognitive impairment. IEEE transactions on audio, speech, and language processing. 2011 Feb 7;19(7):2081–90. doi: 10.1109/TASL.2011.2112351 22199464 PMC3244269

[21] Themistocleous C, Eckerström M, Kokkinakis D. Identification of Mild Cognitive Impairment From Speech in Swedish Using Deep Sequential Neural Networks. Frontiers in Neurology. 2018 Nov 15;9. doi: 10.3389/fneur.2018.00975 30498472 PMC6250092

[22] Themistocleous C, Eckerström M, Kokkinakis D. Voice quality and speech fluency distinguish individuals with Mild Cognitive Impairment from Healthy Controls. Ginsberg SD, editor. PLOS ONE. 2020 Jul 13;15(7):e0236009. doi: 10.1371/journal.pone.0236009 32658934 PMC7357785

[23] Hier DB, Hagenlocker K, Shindler AG. Language disintegration in dementia: Effects of etiology and severity. Brain and Language. 1985 May;25(1):117–33. doi: 10.1016/0093-934x(85)90124-5 2411334

[24] Agis D, Goggins MB, Oishi K, Oishi K, Davis C, Wright A, et al. Picturing the Size and Site of Stroke With an Expanded National Institutes of Health Stroke Scale. Stroke. 2016 Jun;47(6):1459–65. doi: 10.1161/STROKEAHA.115.012324 27217502 PMC4878287

[25] Keator LM, Faria AV, Kim KT, Saxena S, Wright AE, Sheppard SM, et al. An Efficient Bedside Measure Yields Prognostic Implications for Language Recovery in Acute Stroke Patients. Cognitive and Behavioral Neurology. 2020 Sep;33(3):192–200. doi: 10.1097/WNN.0000000000000238 32889951 PMC7479755

[26] Sloane KL, Glenn S, Mefford JA, Zhao Z, Xu M, Zhou G, et al. The validation of a mobile sensor-based neurobehavioral assessment with digital signal processing and machine-learning. Cognitive and Behavioral Neurology. 2022 Sep; 35(3):169–178. 35749748 10.1097/WNN.0000000000000308

[27] Zou H, Hastie T. Regularization and variable selection via the elastic net. Journal of the Royal Statistical Society: Series B (Statistical Methodology). 2005 Apr;67(2):301–20.

[28] Jin C, Choi H, Lee JY. Usefulness of Spontaneous Speech Analysis Scales in Patients with Mild Cognitive Impairment and Dementia of Alzheimer’s Type. Communication Sciences & Disorders. 2016 Jun 8;21(2):284–94.

[29] Bschor T, Kühl KP, Reischies FM. Spontaneous speech of patients with dementia of the Alzheimer type and mild cognitive impairment. Int Psychogeriatr [Internet]. 2001;13(3):289–98. Available from: 10.1017/s1041610201007682 11768376

[30] Knopman DS, Roberts RO, Geda YE, Pankratz VS, Christianson TJH, Petersen RC, et al. Validation of the Telephone Interview for Cognitive Status-modified in Subjects with Normal Cognition, Mild Cognitive Impairment, or Dementia. Neuroepidemiology. 2010;34(1):34–42. doi: 10.1159/000255464 19893327 PMC2857622

[31] Seo EH, Lee DY, Kim SG, Kim KW, Kim DH, Kim B jo, et al. Validity of the telephone interview for cognitive status (TICS) and modified TICS (TICSm) for mild cognitive impairment (MCI) and dementia screening. Archives of Gerontology and Geriatrics. 2011 Jan;52(1):e26–30. 20471701 10.1016/j.archger.2010.04.008

[32] Yesavage JA, Sheikh JI. 9/Geriatric depression scale (GDS) recent evidence and development of a shorter version. Clinical gerontologist. 1986 Nov 18;5(1–2):165–73.

[33] Lezak MD. Relationships between personality disorders, social disturbances, and physical disability following traumatic brain injury. The Journal of head trauma rehabilitation. 1987 Mar.

[34] Malec JF, Lezak MD. 2008. Manual for the Mayo-Portland Adaptability Inventory. Rev ed. http://www.tbims.org/mpai/manual.pdfwww.tbimis.org/combi/mpai. Accessed October 10, 2019.

[35] Folstein MF, Folstein SE, McHugh PR. “Mini-mental state”: a practical method for grading the cognitive state of patients for the clinician. Journal of psychiatric research. 1975 Nov 1;12(3):189–98. 1202204 10.1016/0022-3956(75)90026-6

[36] Chapman KR, Bing-Canar H, Alosco ML, Steinberg EG, Martin B, Chaisson C, et al. Mini Mental State Examination and Logical Memory scores for entry into Alzheimer’s disease trials. Alzheimer’s research & therapy. 2016 Dec;8(1):1–1. doi: 10.1186/s13195-016-0176-z 26899835 PMC4762168

[37] Petersen RC, Lopez O, Armstrong MJ, Getchius TS, Ganguli M, Gloss D, et al. Practice guideline update summary: Mild cognitive impairment: Report of the Guideline Development, Dissemination, and Implementation Subcommittee of the American Academy of Neurology. Neurology. 2018 Jan 16;90(3):126–35. doi: 10.1212/WNL.0000000000004826 29282327 PMC5772157

[38] Nasreddine ZS, Phillips NA, Bedirian V, Charbonneau S, Whitehead V, Collin I, et al. The Montreal Cognitive Assessment, MoCA: A brief screening tool for mild cognitive impairment. Journal of the American Geriatrics Society. 2005 Apr;53(4):695–9. doi: 10.1111/j.1532-5415.2005.53221.x 15817019

[39] Manning C, Surdeanu M, Bauer J, Finkel J, Bethard S, McClosky D. The Stanford CoreNLP natural language processing toolkit. In: Proceedings of 52nd Annual Meeting of the Association for Computational Linguistics: System Demonstrations. Stroudsburg, PA, USA: Association for Computational Linguistics; 2014.

[40] Van Rossum G, Drake FL. (2009). *Python 3 Reference Manual*. Scotts Valley, CA: CreateSpace.

[41] Boersma P. Praat: Doing Phonetics by Computer. 2006. [cited 2021 Apr 18] www.fon.hum.uva.nl. Available from: http://www.praat.org/

[42] Mazumder R, Hastie T, Tibshirani R. Spectral regularization algorithms for learning large incomplete matrices. The Journal of Machine Learning Research. 2010 Aug 1;11:2287–322. 21552465 PMC3087301

[43] Mazumder R, Hastie T. softImpute: Matrix Completion via Iterative Soft-Thresholded SVD [Internet]. R-Packages. 2021 [cited 2022 Apr 6]. Available from: https://cran.r-project.org/web/packages/softImpute/index.html

[44] Adler D, Kelly SD. vioplot: violin plot. R package version 0.3.7 [Internet]. 2021 [cited 2021 Apr 18] Available from: https://github.com/TomKellyGenetics/vioplot

[45] The R Foundation. R: The R Project for Statistical Computing [Internet]. R-project.org. 2019. Available from: https://www.r-project.org/

[46] Benjamini Y, Yekutieli D. The control of the false discovery rate in multiple testing under dependency. Ann Stat [Internet]. 2001;29(4):1165–88. Available from: http://www.jstor.org/stable/2674075

