# Supplementary material for: Varied performance of picture description task as a screening tool across MCI subtypes
Source: PLOS Digit Health. 2023 Mar 13;2(3):e0000197. doi: 10.1371/journal.pdig.0000197 (PMC10010512; doi:10.1371/journal.pdig.0000197)
Supplement: S1 File — (DOCX) [file pdig.0000197.s001.docx]

###

# A. Summary of related studies

### Table A. Studies of separation of participants with mild cognitive impairment from healthy controls using language and speech acoustics features

| **Study** | **Participant counts by subgroup** | **Data type** | **Features extracted** | **Classification approach** | **Performance** |
| --- | --- | --- | --- | --- | --- |
| This study | HC 62, aMCI 18, naMCI 15 | Recordings and transcripts of responses to the PD Cookie Theft task. In English. | Acoustic features from analysis of PD recordings and features for linguistic measures and informative content units in PD transcripts | Measures of classification performance by individual features, by contrasts of pairs of features, and by a classifier built using automatic variable selection and combination of all acoustic and linguistic features with L1-,L2-penalized (“elastic net”) logistic regression | 20 features with p-values below 5e-3 and FDRs at or below 0.113 for association with HC vs aMCI cohort. 61 contrasts of pairs of features with p-values below 5e-4 and FDR at or below 0.132 for association with HC vs aMCI cohort. AUROC 0.88 for classification of HC vs aMCI with penalized logistic regression classifier. |
| Asgari et al, 2017 | MCI 14, HC 27 | Transcripts of unstructured conversations on preselected topics. In English | 68 counts of words in pre-specified word classes | Support vector machines | Baseline AUROC 0.52. AUROC 0.725 using all word features or 0.796 focusing on "relativity" subset of features |
| Bschor et al, 2001 | HC 40, MCI 34, mild DAT 21, moderate to severe DAT 20 | Transcriptions of PD Cookie Theft. In English. | Counts of words and relevant content units with content units partitioned into object, location, feature, and action groups | Classification analysis not used. Anova used to test for statistical differences in word counts across diagnostic groups | DAT described significantly fewer objects and persons, actions, features, and localizations than HC or MCI. No variables differentiated HC and MCI. |
| Calza et al, 2021 | HC 48, aMCI 16, multi-domain MCI 16, early dementia 16 | Recordings and transcripts of semi-spontaneous responses to 3 tasks, including a picture description task (not Cookie Theft). In Italian. | 87 acoustical, rhythmical, morpho-syntactic and lexical features. | SVM to classify combined MCI vs HC. Using all features or manually selected subset. Also p-values for differences in feature distributions across MCI and HC by Kolmagorov-Smirnoff tests. | HC vs MCI classifier F1 measures 0.71 using all features or 0.74 using a curated set of features. |
| Eyigoz et al, 2020 | 270 HC at time of assessment. Half of the study participants subsequently were diagnosed with AD on or before an age of 85 years | Written responses to PD Cookie Theft. In English. | 87 linguistic features based on written responses | Logistic regression after variable selection. In addition to linguistic features, demographic and clinical covariates and scores from a battery of 13 neuropsychological tests besides Picture Description were used in the predictive modeling. | AUROC in hold-out test set for prediction of diagnosis of AD by age 85 0.74 using linguistic features and 0.67 using an aggregate of linguistic and non-linguistic features. AUROC 0.60 using only non-linguistic variables. |
| Fraser et al, 2019 | HC 29, MCI 26 | Recordings, transcripts, and eye tracking features from PD Cookie Theft and two reading tasks. In Swedish. | 26 language features, 12 speech or acoustics features, plus eye tracking features and results of reading comprehension tests | SVM with non-linear kernels (radial basis function) and L2-penalized logistic regression | AUC 0.71 using combined features from Picture description (language and speech) and either SVM or logistic regression. AUC 0.88 using all tasks and features and either SVM or logistic regression. AUC 0.40 for SVM with speech features only. AUC 0.73 for SVM with language features only. |
| Gosztolya et al, 2019 | HC 25, MCI 25, AD 25 | Recordings of spontaneous speech, in Hungarian | Acoustic or phonetic features from custom neural network analysis of recordings and language features from analysis of transcripts | Support vector machines. Demographic variables included in models. | HC vs MCI: F1 56.5 using demographic features; F1 0.756 adding acoustic features; F1 0.727 adding language features; F1 0.783 adding acoustic and language features;F1 0.857 adding acoustic and semantic-language features |
| Hernández-Domínguez et al, 2018 | 25 HC in reference analysis. 217 HC, 257 AD, and 43 MCI in classification analysis | Recordings and transcripts of PD Cookie Theft task. In English. | Mel-frequency cepstral coefficients from recordings and language features extracted from PD transcripts | SVM with phonetic, language, and other "information coverage" features extracted from PD | Performance varied with use of the phonetic, language, or coverage features as inputs to the SVM. Best average performance with linguistic + coverage features: AUROC 0.79 HC vs AD; 0.76 HC vs (AD+MCI) |
| Jin et al, 2016 | DAT 30, MCI 30, HC 30 | Transcripts from PD Cookie Theft. In Korean | Ratio of Correct Information Unit (CIU) to morphological words, the ratio of CIU to syntactic words, main concepts, and the number of content words by utterance. | Tests of statistical difference in values of features rather than assessment of classification performance | HC had significantly higher performance than the MCI group only in the ratio of content words to utterance |
| König et al, 2015 | HC 15, MCI 23, AD 26 | Recordings of 4 tasks: picture description, counting backwards, sentence repetition, semantic fluency (mammals) | Vocal features based on lengths and periodicity of voiced and silent segments in recordings | Pairwise analyses of diagnoses. Classification with SVM after feature selection by significance threshold on Mann-Whitney test for association with diagnosis | Accuracy:  HC vs MCI 0.79  HC vs AD 0.87  MCI vs AD 0.80 |
| Roark et al, 2011 | HC 37, MCI 37 | Recordings and transcripts from study participants retelling a three-sentence story. Two retellings: one immediately after hearing the story; the other after 30 minutes of unrelated activities | Timestamps of words and pauses aligned to transcripts. Measures of speech production rate, pauses, and alignment with original story calculated. | Language and speech feature selection by marginal association test for association with diagnosis. Best performing SVM uses selected features plus results from 9 neuropsych tests | AUROCs for SVM classifiers using different feature sets:  Individually significant speech and language features: 0.703  Using 9 neuropsych test scores: 0.815  Significant features + 9 neuropsych test scores: 0.861 |
| Themistoc leous et al, 2018 | HC 30, MCI 25 | Recordings of participants reading a 144 word passage. In Swedish. | Each vowel was identified and segmented from the recording, and processed to provide: duration, F0, and formants F1-F5 | Classification using neural networks with 1-10 hidden layers | Accuracy (5-fold cv) 0.82 with 4 or 5 hidden layers; 0.83 with 6 or more hidden layers. Hold-out set accuracy (90-10 split) 0.75 with 7 hidden layers. |
| Themistoc leous et al, 2020 | MCI 26, HC 29 | Recordings of PD Cookie Theft. In Swedish | Acoustic features and also counts and rates of syllables | Tests of statistical differences in values of features across diagnostic groups and of regression coefficients for diagnosis in regression models for features rather than assessment of classification performance | Syllable duration, articulation rate, shimmer, cepstral peak prominence, and difference of the first harmonic and third amplitude were statistically significantly different between HC and MCI groups |

**PD** = picture description assessment task. **HC** = healthy control. **MCI** = mild cognitive impairment. **AD** = Alzheimer’s Disease. **DAT** = dementia of Alzheimer’s type. **SVM** = support vector machine. **AUROC** = area under the receiver operator characteristic curve. **F1** = measure of classifier performance. **FDR** = Benjamini-Yakutieli false discovery rate.

# B. Miro Health Picture Description features by diagnostic cohort

### Table B. Participant features raw scores

| **Variable** | **HC**  **Mean (sd)** | **aMCI**  **Mean (sd)** | **naMCI**  **Mean (sd)** |
| --- | --- | --- | --- |
| Acoustic APQ (%) | 9.62 (2.22) | 10.7 (2.4) | 10.6 (2.9) |
| Acoustic ATRI (%) or ATrI | 43.1 (12.1) | 32.8 (14.4) | 45.5 (8.5) |
| Acoustic ATrP | 31.3 (6.6) | 26.1 (12.0) | 32.9 (6.1) |
| Acoustic DDP | 3.35 (1.24) | 3.61 (1.49) | 3.68 (1.22) |
| Acoustic DUV | 52.5 (13.3) | 61.5 (16.9) | 46.8 (12.7) |
| Acoustic DVB (%) | 51.0 (10.8) | 55.4 (10.1) | 46.5 (13.2) |
| Acoustic F0 (Hz) | 151 (29.0) | 153 (42.0) | 138 (25.0) |
| Acoustic F0spread16to50 | 24.2 (12.8) | 26.6 (13.9) | 24.4 (18.2) |
| Acoustic F0spread50to84 | 25.1 (21.1) | 36.9 (31.4) | 28.5 (29.7) |
| Acoustic F1 | 630 (194) | 699 (195) | 662 (161) |
| Acoustic F2 | 1.7e+03 (3.1e+02) | 1.9e+03 (2.3e+02) | 1.8e+03 (2.5e+02) |
| Acoustic F3 | 2.7e+03 (4.1e+02) | 2.9e+03 (3.2e+02) | 2.8e+03 (3.6e+02) |
| Acoustic F4 | 3.7e+03 (5.7e+02) | 3.9e+03 (4.5e+02) | 3.8e+03 (4.7e+02) |
| Acoustic F5 | 4.4e+03 (5.6e+02) | 4.7e+03 (4.4e+02) | 4.6e+03 (4.9e+02) |
| Acoustic Fatr (Hz) or ATrF | 3.65 (2.53) | 5.41 (4.64) | 3.19 (1.94) |
| Acoustic Fftr (Hz) or FTrF | 4.34 (2.46) | 5.7 (4.23) | 6.86 (4.95) |
| Acoustic Fhi (Hz) | 474 (138) | 537 (83.0) | 475 (92.0) |
| Acoustic Flo (Hz) | 72.1 (7.7) | 71.3 (3.4) | 71.7 (3.8) |
| Acoustic FTRI (%) or FTrI | 18.9 (10.4) | 21.0 (17.2) | 15.7 (5.5) |
| Acoustic FTrP | 14.4 (7.7) | 15.4 (10.7) | 13.0 (4.7) |
| Acoustic Harmonicity_10p | 5.72 (3.13) | 3.9 (2.67) | 4.63 (2.54) |
| Acoustic Harmonicity_50p | 14.4 (3.2) | 13.4 (2.8) | 13.5 (3.2) |
| Acoustic Harmonicity_90p | 21.2 (3.6) | 20.9 (3.1) | 20.1 (3.4) |
| Acoustic Harmonicity_Average | 13.9 (3.1) | 12.9 (2.7) | 12.9 (3.0) |
| Acoustic Harmonicity_Stdev | 6.01 (1.03) | 6.49 (0.85) | 5.93 (0.71) |
| Acoustic Jita (s) | - (-) | - (-) | - (-) |
| Acoustic Jitt (%) | 2.4 (0.75) | 2.61 (0.8) | 2.62 (0.75) |
| Acoustic Mean_absolute_slope | 296 (117) | 316 (204) | 323 (112) |
| Acoustic NHR | 14.8 (5.9) | 17.1 (5.3) | 16.7 (6.8) |
| Acoustic NUV | 1.4e+04 (3.6e+03) | 1.6e+04 (5.2e+03) | 1.2e+04 (3.6e+03) |
| Acoustic NVB (%) | 163 (70.0) | 116 (54.0) | 160 (42.0) |
| Acoustic PFR | 30.3 (6.6) | 33.4 (3.0) | 31.0 (3.6) |
| Acoustic PPQ (%) | 1.14 (0.33) | 1.24 (0.44) | 1.25 (0.4) |
| Acoustic RAP (%) | 1.12 (0.41) | 1.2 (0.5) | 1.23 (0.41) |
| Acoustic Shdb (dB) | 1.03 (0.21) | 1.09 (0.19) | 1.1 (0.23) |
| Acoustic Shim (%) | 10.6 (2.7) | 11.2 (2.4) | 11.3 (3.2) |
| Acoustic Shimmer (apq3) | 4.72 (1.82) | 4.9 (1.51) | 5.1 (2.09) |
| Acoustic Shimmer (apq5) | 6.18 (2.2) | 6.64 (2.04) | 6.77 (2.61) |
| Acoustic Shimmer (dda) | 14.2 (5.5) | 14.7 (4.5) | 15.3 (6.3) |
| Acoustic SPI | 84.9 (89.3) | 71.8 (68.2) | 80.1 (55.0) |
| Acoustic Tsam (sec) | 90.1 (0.1) | 85.6 (13.0) | 87.3 (7.4) |
| Acoustic VTI | 0.01 (0.01) | 0.01 (0.01) | 0.01 (0.01) |
| Acoustics standard deviation of F0 | 36.1 (14.9) | 47.1 (20.1) | 35.0 (14.9) |
| Acoustics voice breaks percentage | 2.4e+03 (1.1e+03) | 3.1e+03 (9.3e+02) | 2.5e+03 (9.4e+02) |
| Auxiliary Verbs | 8.92 (4.81) | 6.28 (5.9) | 9.07 (3.61) |
| Both Sides Total Content Units | 13.3 (9.1) | 3.44 (3.68) | 10.1 (8.6) |
| Both Sides Total Content Units Unique | 8.16 (4.4) | 2.56 (2.59) | 6.47 (4.87) |
| Conjunction | 7.85 (5.59) | 5.11 (5.98) | 7.6 (4.85) |
| Count of ALL content units | 86.9 (25.6) | 53.7 (24.2) | 79.9 (23.6) |
| Count of ALL DISTINCT content units | 58.8 (15.7) | 40.1 (16.6) | 55.3 (13.9) |
| Count of all function words | 77.1 (28.1) | 50.6 (34.8) | 75.3 (19.6) |
| Count of complete sentences | 10.8 (5.8) | 5.56 (5.55) | 9.6 (6.47) |
| Count of content units on LEFT side of picture | 18.1 (5.1) | 13.3 (5.5) | 17.3 (4.4) |
| Count of content units on RIGHT side of picture | 28.6 (7.1) | 19.4 (7.9) | 25.3 (6.7) |
| Count of difference words: other, another | 0.19 (0.47) | 0.11 (0.32) | 0.2 (0.56) |
| Count of DISTINCT content units on Left side of picture | 12.0 (2.7) | 9.33 (3.4) | 11.5 (2.6) |
| Count of DISTINCT content units on RIGHT side of picture | 20.5 (4.5) | 15.1 (5.5) | 18.2 (4.3) |
| Count of DISTINCT RELATED content units | 40.5 (9.1) | 26.9 (9.2) | 36.1 (10.3) |
| Count of distributive determiners | 0.48 (1.11) | 0.44 (0.78) | 0.27 (0.46) |
| Count of indefinite articles | 8.03 (5.37) | 2.11 (2.49) | 6.13 (3.6) |
| Count of modals | 0.18 (0.5) | 0.33 (0.59) | 0.33 (0.62) |
| Count of nouns | 46.5 (11.7) | 30.2 (11.5) | 41.9 (12.3) |
| Count of phrases | 125 (39.0) | 84.4 (43.1) | 118 (29.0) |
| Count of pre-determiners | 0.06 (0.4) | 0.11 (0.47) | 0.0 (0.0) |
| Count of prepositions | 16.6 (5.8) | 8.56 (6.1) | 14.0 (5.4) |
| Count of pronouns | 6.0 (5.14) | 4.61 (4.73) | 5.6 (3.07) |
| Count of quantifiers | 1.71 (2.35) | 1.17 (1.76) | 1.47 (1.41) |
| Count of RELATED content units | 60.0 (16.1) | 36.2 (13.6) | 52.7 (17.1) |
| Count of sentences | 15.2 (5.7) | 9.83 (5.27) | 13.3 (5.8) |
| Count of Syllables | 215 (63.0) | 136 (68.0) | 199 (50.0) |
| Count of utterances | 3.82 (3.3) | 4.56 (3.84) | 5.4 (3.85) |
| Count of words | 166 (52.0) | 103 (57.0) | 157 (41.0) |
| Definite Article | 20.7 (8.0) | 15.4 (8.4) | 21.9 (8.0) |
| Demonstrative | 1.5 (1.46) | 0.83 (1.58) | 1.07 (0.88) |
| Number of Adverbs | 4.39 (3.61) | 2.94 (3.32) | 5.13 (4.85) |
| Number of Verbs | 33.8 (12.1) | 24.8 (15.8) | 33.9 (7.7) |
| Numbers | 1.63 (2.48) | 0.33 (0.69) | 1.87 (3.74) |
| Particles | 3.39 (2.73) | 2.94 (2.69) | 4.0 (2.7) |
| Possessive Determiners | 1.19 (1.4) | 0.83 (1.15) | 0.93 (0.96) |
| Pro-sentences | 0.06 (0.25) | 0.22 (0.43) | 0.0 (0.0) |
| Qualifiers | 0.29 (0.69) | 0.17 (0.51) | 0.27 (0.59) |
| Question Words | 1.16 (1.32) | 1.0 (2.17) | 1.47 (1.81) |
| SD Number of Syllables / Word | 0.5 (0.06) | 0.52 (0.08) | 0.51 (0.08) |
| Syllables: Utterances Ratio | 102 (75.0) | 54.5 (49.8) | 66.4 (54.0) |
| Syllables: Word Ratio (mean) | 1.3 (0.07) | 1.38 (0.18) | 1.28 (0.08) |
| Syllables per word max | 3.31 (0.69) | 3.17 (0.62) | 3.33 (0.82) |
| Syllables per word min | 1.0 (0.0) | 1.0 (0.0) | 1.0 (0.0) |
| Total Content Units / Total Words | 0.53 (0.06) | 0.57 (0.14) | 0.51 (0.05) |
| Total Function Words / Content Units | 0.89 (0.2) | 0.87 (0.42) | 0.97 (0.21) |
| Total Function Words / Total Words | 0.46 (0.06) | 0.45 (0.15) | 0.48 (0.05) |
| Variability (vector distance) | 4.74 (1.92) | 4.86 (3.56) | 5.3 (1.57) |

**HC** = healthy control. **aMCI** = amnestic mild cognitive impairment.  **naMCI** = nonamnestic mild cognitive impairment.

### Table C. Participant features Quantile Normalized scores

| **Variable** | **HC**  **Mean (sd)** | **aMCI**  **Mean (sd)** | **naMCI**  **Mean (sd)** |
| --- | --- | --- | --- |
| Acoustic APQ (%) | -0.14 (0.91) | 0.25 (0.92) | 0.21 (1.14) |
| Acoustic ATRI (%) or ATrI | 0.09 (0.97) | -0.6 (0.88) | 0.32 (0.62) |
| Acoustic ATrP | 0.04 (0.93) | -0.36 (1.16) | 0.21 (0.82) |
| Acoustic DDP | -0.08 (0.94) | 0.05 (1.09) | 0.2 (0.89) |
| Acoustic DUV | -0.05 (0.89) | 0.49 (1.05) | -0.48 (0.9) |
| Acoustic DVB (%) | -0.02 (0.9) | 0.35 (0.89) | -0.43 (1.16) |
| Acoustic F0 (Hz) | 0.03 (0.94) | 0.05 (1.16) | -0.29 (0.75) |
| Acoustic F0spread16to50 | -0.03 (0.97) | 0.14 (1.04) | -0.15 (0.84) |
| Acoustic F0spread50to84 | -0.1 (0.92) | 0.31 (1.07) | -0.06 (0.95) |
| Acoustic F1 | -0.11 (0.97) | 0.26 (1.03) | 0.05 (0.82) |
| Acoustic F2 | -0.11 (1.03) | 0.32 (0.75) | -0.01 (0.84) |
| Acoustic F3 | -0.16 (0.98) | 0.43 (0.77) | 0.04 (0.94) |
| Acoustic F4 | -0.11 (1.0) | 0.29 (0.87) | -0.0 (0.85) |
| Acoustic F5 | -0.11 (0.98) | 0.21 (0.76) | 0.09 (1.07) |
| Acoustic Fatr (Hz) or ATrF | -0.07 (0.9) | 0.48 (0.88) | -0.42 (1.24) |
| Acoustic Fftr (Hz) or FTrF | -0.14 (0.89) | 0.12 (1.12) | 0.53 (1.06) |
| Acoustic Fhi (Hz) | -0.1 (1.02) | 0.42 (0.82) | -0.18 (0.7) |
| Acoustic Flo (Hz) | -0.03 (1.0) | -0.01 (0.84) | 0.05 (0.97) |
| Acoustic FTRI (%) or FTrI | 0.01 (0.92) | -0.02 (1.37) | -0.14 (0.48) |
| Acoustic FTrP | -0.02 (0.95) | 0.0 (1.23) | 0.04 (0.64) |
| Acoustic Harmonicity_10p | 0.15 (0.98) | -0.42 (0.89) | -0.19 (0.81) |
| Acoustic Harmonicity_50p | 0.09 (0.98) | -0.21 (0.84) | -0.22 (1.01) |
| Acoustic Harmonicity_90p | 0.07 (0.98) | -0.07 (0.87) | -0.29 (0.97) |
| Acoustic Harmonicity_Average | 0.09 (0.98) | -0.21 (0.87) | -0.23 (0.98) |
| Acoustic Harmonicity_Stdev | -0.09 (1.01) | 0.38 (0.86) | -0.19 (0.76) |
| Acoustic Jita (s) | -0.12 (0.96) | 0.09 (1.04) | 0.27 (0.84) |
| Acoustic Jitt (%) | -0.12 (0.96) | 0.19 (0.9) | 0.19 (1.02) |
| Acoustic Mean_absolute_slope | -0.05 (0.94) | -0.07 (1.16) | 0.19 (0.81) |
| Acoustic NHR | -0.14 (0.98) | 0.26 (0.82) | 0.18 (0.99) |
| Acoustic NUV | 0.02 (0.84) | 0.3 (1.21) | -0.52 (0.94) |
| Acoustic NVB (%) | 0.12 (0.96) | -0.6 (0.94) | 0.15 (0.76) |
| Acoustic PFR | -0.1 (1.02) | 0.41 (0.8) | -0.19 (0.72) |
| Acoustic PPQ (%) | -0.09 (0.91) | 0.11 (0.99) | 0.16 (1.15) |
| Acoustic RAP (%) | -0.08 (0.94) | 0.05 (1.09) | 0.2 (0.89) |
| Acoustic Shdb (dB) | -0.11 (0.98) | 0.18 (0.88) | 0.17 (0.96) |
| Acoustic Shim (%) | -0.11 (0.98) | 0.18 (0.86) | 0.13 (0.98) |
| Acoustic Shimmer (apq3) | -0.08 (1.01) | 0.09 (0.86) | 0.13 (0.9) |
| Acoustic Shimmer (apq5) | -0.1 (0.98) | 0.14 (0.89) | 0.15 (0.97) |
| Acoustic Shimmer (dda) | -0.08 (1.01) | 0.09 (0.86) | 0.13 (0.9) |
| Acoustic SPI | 0.01 (0.97) | -0.08 (0.91) | 0.13 (0.87) |
| Acoustic Tsam (sec) | 0.18 (0.86) | -0.41 (1.09) | -0.38 (0.95) |
| Acoustic VTI | -0.03 (0.96) | 0.03 (0.87) | -0.17 (0.99) |
| Acoustics standard deviation of F0 | -0.12 (0.92) | 0.5 (0.9) | -0.18 (1.02) |
| Acoustics voice breaks percentage | -0.16 (0.95) | 0.53 (0.76) | -0.06 (1.05) |
| Auxiliary Verbs | 0.07 (0.92) | -0.37 (1.16) | 0.06 (0.72) |
| Both Sides Total Content Units | 0.23 (0.93) | -0.85 (0.66) | -0.03 (0.81) |
| Both Sides Total Content Units Unique | 0.23 (0.91) | -0.88 (0.64) | -0.01 (0.9) |
| Conjunction | 0.08 (0.94) | -0.44 (1.01) | 0.04 (0.91) |
| Count of ALL content units | 0.23 (0.92) | -0.85 (0.75) | -0.03 (0.83) |
| Count of ALL DISTINCT content units | 0.2 (0.91) | -0.79 (0.88) | 0.02 (0.8) |
| Count of all function words | 0.16 (0.9) | -0.7 (1.12) | 0.1 (0.65) |
| Count of complete sentences | 0.17 (0.9) | -0.68 (0.88) | 0.01 (1.0) |
| Count of content units on LEFT side of picture | 0.16 (0.92) | -0.66 (0.95) | 0.03 (0.84) |
| Count of content units on RIGHT side of picture | 0.26 (0.89) | -0.82 (0.83) | -0.17 (0.87) |
| Count of difference words: other, another | -0.0 (0.65) | -0.11 (0.52) | -0.03 (0.69) |
| Count of DISTINCT content units on Left side of picture | 0.16 (0.91) | -0.64 (0.98) | -0.01 (0.89) |
| Count of DISTINCT content units on RIGHT side of picture | 0.24 (0.89) | -0.73 (0.91) | -0.24 (0.82) |
| Count of DISTINCT RELATED content units | 0.27 (0.86) | -0.93 (0.7) | -0.11 (1.0) |
| Count of distributive determiners | -0.02 (0.79) | 0.04 (0.78) | -0.1 (0.65) |
| Count of indefinite articles | 0.26 (0.91) | -0.94 (0.68) | -0.04 (0.73) |
| Count of modals | -0.1 (0.6) | 0.12 (0.77) | 0.11 (0.78) |
| Count of nouns | 0.25 (0.9) | -0.89 (0.7) | -0.07 (0.91) |
| Count of phrases | 0.19 (0.91) | -0.74 (0.97) | -0.01 (0.73) |
| Count of pre-determiners | -0.02 (0.38) | 0.02 (0.48) | -0.09 (0.0) |
| Count of prepositions | 0.26 (0.85) | -0.92 (0.91) | -0.1 (0.8) |
| Count of pronouns | 0.02 (1.02) | -0.25 (0.96) | 0.12 (0.63) |
| Count of quantifiers | 0.03 (0.94) | -0.24 (0.88) | 0.06 (0.83) |
| Count of RELATED content units | 0.29 (0.87) | -0.95 (0.65) | -0.13 (0.93) |
| Count of sentences | 0.19 (0.9) | -0.7 (0.91) | -0.08 (0.92) |
| Count of Syllables | 0.23 (0.91) | -0.82 (0.88) | -0.04 (0.73) |
| Count of utterances | -0.12 (0.89) | 0.05 (1.02) | 0.34 (0.91) |
| Count of words | 0.21 (0.92) | -0.81 (0.91) | -0.0 (0.7) |
| Definite Article | 0.09 (0.93) | -0.53 (0.95) | 0.17 (0.94) |
| Demonstrative | 0.12 (0.91) | -0.4 (0.93) | -0.09 (0.69) |
| Number of Adverbs | 0.05 (0.9) | -0.43 (0.97) | 0.21 (1.04) |
| Number of Verbs | 0.11 (0.93) | -0.53 (1.17) | 0.08 (0.61) |
| Numbers | 0.09 (0.9) | -0.51 (0.56) | 0.15 (0.85) |
| Particles | -0.01 (0.93) | -0.26 (1.08) | 0.21 (0.86) |
| Possessive Determiners | 0.05 (0.9) | -0.22 (0.91) | -0.07 (0.78) |
| Pro-sentences | -0.06 (0.46) | 0.23 (0.8) | -0.18 (0.0) |
| Qualifiers | -0.0 (0.69) | -0.13 (0.55) | 0.01 (0.67) |
| Question Words | 0.02 (0.85) | -0.25 (0.88) | 0.14 (0.97) |
| SD Number of Syllables / Word | -0.04 (0.83) | 0.13 (1.23) | -0.09 (1.16) |
| Syllables: Utterances Ratio | 0.21 (0.9) | -0.56 (0.97) | -0.31 (0.88) |
| Syllables: Word Ratio (mean) | -0.02 (0.85) | 0.32 (1.14) | -0.42 (1.05) |
| Syllables per word max | 0.0 (0.82) | -0.15 (0.83) | 0.02 (1.03) |
| Syllables per word min | -0.03 (-) | -0.03 (-) | -0.03 (-) |
| Total Content Units / Total Words | -0.0 (0.81) | 0.18 (1.41) | -0.28 (0.89) |
| Total Function Words / Content Units | -0.05 (0.81) | -0.1 (1.43) | 0.25 (0.87) |
| Total Function Words / Total Words | -0.07 (0.81) | -0.03 (1.45) | 0.25 (0.84) |
| Variability (vector distance) | -0.04 (0.88) | -0.18 (1.26) | 0.3 (0.86) |

**HC** = healthy control. **aMCI** = amnestic mild cognitive impairment. **naMCI** = nonamnestic mild cognitive impairment.

# C. Association of Miro Health Picture Description features with MCI subtypes

### Table D. Association of features derived from picture description recordings or transcripts with diagnosis of aMCI or HC

| **Variable** | **p-value** | **FDR** | **AUROC** | **Effect Direction** |
| --- | --- | --- | --- | --- |
| Count of DISTINCT RELATED content units | 1.1E-04 | 0.019 | 0.82 | (-) |
| Count of RELATED content units | 1.2E-04 | 0.019 | 0.83 | (-) |
| Count of indefinite articles | 1.7E-04 | 0.019 | 0.82 | (-) |
| Count of prepositions | 2.1E-04 | 0.019 | 0.79 | (-) |
| Count of nouns | 2.3E-04 | 0.019 | 0.81 | (-) |
| Both Sides Total Content Units Unique | 2.5E-04 | 0.019 | 0.80 | (-) |
| Both Sides Total Content Units | 3.3E-04 | 0.021 | 0.78 | (-) |
| Count of ALL content units | 3.7E-04 | 0.021 | 0.76 | (-) |
| Count of content units on RIGHT side of picture | 4.1E-04 | 0.021 | 0.75 | (-) |
| Count of Syllables | 6.0E-04 | 0.028 | 0.74 | (-) |
| Count of words | 7.9E-04 | 0.032 | 0.73 | (-) |
| Count of ALL DISTINCT content units | 8.2E-04 | 0.032 | 0.73 | (-) |
| Count of DISTINCT content units on RIGHT side of picture | 1.1E-03 | 0.039 | 0.71 | (-) |
| Count of sentences | 1.5E-03 | 0.049 | 0.69 | (-) |
| Count of phrases | 1.6E-03 | 0.049 | 0.71 | (-) |
| Acoustics standard deviation of F0 | 2.8E-03 | 0.080 | 0.71 | + |
| Count of complete sentences | 4.3E-03 | 0.113 | 0.66 | (-) |
| Count of content units on LEFT side of picture | 4.4E-03 | 0.113 | 0.66 | (-) |
| Count of all function words | 4.9E-03 | 0.113 | 0.67 | (-) |
| Count of DISTINCT content units on Left side of picture | 4.9E-03 | 0.113 | 0.65 | (-) |
| Acoustic F0spread50to84 | 5.9E-03 | 0.13 | 0.61 | + |
| Syllables: Utterances Ratio | 6.6E-03 | 0.14 | 0.64 | (-) |
| Acoustics voice breaks percentage | 8.3E-03 | 0.17 | 0.67 | + |
| Acoustic NVB (%) | 0.020 | 0.38 | 0.61 | (-) |
| Numbers | 0.022 | 0.40 | 0.61 | (-) |
| Acoustic Tsam (sec) | 0.029 | 0.51 | 0.57 | (-) |
| Acoustic F3 | 0.031 | 0.52 | 0.58 | + |
| Count of modals | 0.036 | 0.60 | 0.55 | + |
| Definite Article | 0.039 | 0.62 | 0.56 | (-) |
| Number of Verbs | 0.044 | 0.69 | 0.60 | (-) |
| Acoustic DUV | 0.051 | 0.76 | 0.59 | + |
| Pro-sentences | 0.056 | 0.80 | 0.55 | + |
| Acoustic Harmonicity_Stdev | 0.057 | 0.80 | 0.57 | + |
| Acoustic PFR | 0.062 | 0.85 | 0.56 | + |
| Acoustic Fhi (Hz) | 0.069 | 0.90 | 0.56 | + |
| Acoustic Harmonicity_10p | 0.070 | 0.90 | 0.55 | (-) |
| Demonstrative | 0.11 | 1.00 | 0.57 | (-) |
| Acoustic ATRI (%) or ATrI | 0.11 | 1.00 | 0.56 | (-) |
| Conjunction | 0.11 | 1.00 | 0.57 | (-) |
| Acoustic F0spread16to50 | 0.13 | 1.00 | 0.56 | + |
| Number of Adverbs | 0.13 | 1.00 | 0.55 | (-) |
| Acoustic F4 | 0.13 | 1.00 | 0.54 | + |
| Acoustic F2 | 0.14 | 1.00 | 0.54 | + |
| Acoustic Fftr (Hz) or FTrF | 0.14 | 1.00 | 0.55 | + |
| Acoustic F0 (Hz) | 0.15 | 1.00 | 0.56 | + |
| Acoustic ATrP | 0.17 | 1.00 | 0.55 | (-) |
| Acoustic NHR | 0.18 | 1.00 | 0.54 | + |
| Acoustic DVB (%) | 0.18 | 1.00 | 0.53 | + |
| Auxiliary Verbs | 0.19 | 1.00 | 0.54 | (-) |
| Acoustic F5 | 0.19 | 1.00 | 0.54 | + |
| Acoustic APQ (%) | 0.27 | 1.00 | 0.55 | + |
| Acoustic F1 | 0.28 | 1.00 | 0.54 | + |
| Syllables: Word Ratio (mean) | 0.28 | 1.00 | 0.54 | + |
| Acoustic NUV | 0.30 | 1.00 | 0.54 | + |
| Particles | 0.32 | 1.00 | 0.55 | (-) |
| Acoustic Shdb (dB) | 0.36 | 1.00 | 0.56 | + |
| Acoustic Shim (%) | 0.37 | 1.00 | 0.55 | + |
| Question Words | 0.39 | 1.00 | 0.53 | (-) |
| Syllables per word max | 0.40 | 1.00 | 0.54 | (-) |
| Acoustic Jitt (%) | 0.40 | 1.00 | 0.55 | + |
| Acoustic Shimmer (apq5) | 0.41 | 1.00 | 0.56 | + |
| Possessive Determiners | 0.42 | 1.00 | 0.55 | (-) |
| Acoustic Harmonicity_Average | 0.42 | 1.00 | 0.54 | (-) |
| Count of quantifiers | 0.42 | 1.00 | 0.53 | (-) |
| Acoustic Harmonicity_50p | 0.45 | 1.00 | 0.55 | (-) |
| Acoustic Fatr (Hz) or ATrF | 0.48 | 1.00 | 0.55 | + |
| Acoustic Shimmer (apq3) | 0.52 | 1.00 | 0.57 | + |
| Acoustic Shimmer (dda) | 0.52 | 1.00 | 0.56 | + |
| Count of distributive determiners | 0.53 | 1.00 | 0.57 | + |
| Count of utterances | 0.55 | 1.00 | 0.56 | + |
| Count of difference words: other, another | 0.55 | 1.00 | 0.53 | (-) |
| Count of pronouns | 0.55 | 1.00 | 0.54 | (-) |
| Acoustic Mean_absolute_slope | 0.57 | 1.00 | 0.56 | + |
| Acoustic FTRI (%) or FTrI | 0.57 | 1.00 | 0.56 | (-) |
| SD Number of Syllables / Word | 0.57 | 1.00 | 0.55 | + |
| Count of pre-determiners | 0.59 | 1.00 | 0.55 | + |
| Qualifiers | 0.60 | 1.00 | 0.54 | (-) |
| Total Function Words / Total Words | 0.61 | 1.00 | 0.59 | + |
| Acoustic Flo (Hz) | 0.62 | 1.00 | 0.55 | (-) |
| Acoustic PPQ (%) | 0.64 | 1.00 | 0.58 | + |
| Acoustic FTrP | 0.72 | 1.00 | 0.57 | (-) |
| Acoustic RAP (%) | 0.78 | 1.00 | 0.59 | + |
| Acoustic DDP | 0.78 | 1.00 | 0.59 | + |
| Variability (vector distance) | 0.79 | 1.00 | 0.57 | (-) |
| Syllables per word min | 0.81 | 1.00 | 0.54 | (-) |
| Total Content Units / Total Words | 0.81 | 1.00 | 0.57 | + |
| Total Function Words / Content Units | 0.85 | 1.00 | 0.59 | + |
| Acoustic Harmonicity_90p | 0.85 | 1.00 | 0.57 | (-) |
| Acoustic VTI | 0.92 | 1.00 | 0.56 | + |
| Acoustic SPI | 0.99 | 1.00 | 0.55 | + |
| Acoustic Jita (s) | 1.00 | 1.00 | 0.57 | + |

**HC** = healthy control. **aMCI** = amnestic mild cognitive impairment.  **naMCI** = nonamnestic mild cognitive impairment. **FDR** = Benjamini Yakutieli false discovery rate. **AUROC** = area under the receiver operator curve.

### Table E. Association of features derived from picture description recordings or transcripts with diagnosis of naMCI or HC

###

| **Variable** | **p-value** | **FDR** | **AUROC** | **Effect Direction** |
| --- | --- | --- | --- | --- |
| Acoustic Tsam (sec) | 0.036 | 1 | 0.57 | (-) |
| Acoustic NUV | 0.044 | 1 | 0.59 | (-) |
| Count of DISTINCT content units on RIGHT side of picture | 0.066 | 1 | 0.58 | (-) |
| Syllables: Utterances Ratio | 0.085 | 1 | 0.55 | (-) |
| Syllables: Word Ratio (mean) | 0.086 | 1 | 0.58 | (-) |
| Count of RELATED content units | 0.11 | 1 | 0.58 | (-) |
| Count of content units on RIGHT side of picture | 0.11 | 1 | 0.57 | (-) |
| Total Function Words / Total Words | 0.12 | 1 | 0.58 | + |
| Count of DISTINCT RELATED content units | 0.12 | 1 | 0.57 | (-) |
| Count of prepositions | 0.12 | 1 | 0.58 | (-) |
| Acoustic DUV | 0.12 | 1 | 0.57 | (-) |
| Total Function Words / Content Units | 0.14 | 1 | 0.58 | + |
| Count of utterances | 0.15 | 1 | 0.55 | + |
| Acoustic DVB (%) | 0.15 | 1 | 0.56 | (-) |
| Variability (vector distance) | 0.15 | 1 | 0.57 | + |
| Count of indefinite articles | 0.16 | 1 | 0.58 | (-) |
| Total Content Units / Total Words | 0.16 | 1 | 0.59 | (-) |
| Count of modals | 0.17 | 1 | 0.57 | + |
| Count of nouns | 0.19 | 1 | 0.57 | (-) |
| Acoustic Mean_absolute_slope | 0.21 | 1 | 0.57 | + |
| Acoustic Harmonicity_90p | 0.22 | 1 | 0.59 | (-) |
| Acoustic Fftr (Hz) or FTrF | 0.22 | 1 | 0.59 | + |
| Count of sentences | 0.24 | 1 | 0.59 | (-) |
| Both Sides Total Content Units | 0.26 | 1 | 0.60 | (-) |
| Both Sides Total Content Units Unique | 0.27 | 1 | 0.59 | (-) |
| Acoustic Harmonicity_10p | 0.27 | 1 | 0.58 | (-) |
| Count of Syllables | 0.27 | 1 | 0.59 | (-) |
| Acoustic NHR | 0.29 | 1 | 0.59 | + |
| Acoustic Harmonicity_Average | 0.29 | 1 | 0.59 | (-) |
| Count of ALL content units | 0.30 | 1 | 0.58 | (-) |
| Acoustic APQ (%) | 0.30 | 1 | 0.59 | + |
| Acoustic Jita (s) | 0.32 | 1 | 0.57 | + |
| Acoustic Harmonicity_50p | 0.32 | 1 | 0.58 | (-) |
| Acoustic Shdb (dB) | 0.37 | 1 | 0.60 | + |
| Particles | 0.37 | 1 | 0.60 | + |
| Count of words | 0.38 | 1 | 0.59 | (-) |
| Acoustic F0spread50to84 | 0.39 | 1 | 0.59 | + |
| Count of phrases | 0.40 | 1 | 0.58 | (-) |
| Acoustic Jitt (%) | 0.40 | 1 | 0.58 | + |
| Count of ALL DISTINCT content units | 0.41 | 1 | 0.59 | (-) |
| Count of DISTINCT content units on Left side of picture | 0.42 | 1 | 0.60 | (-) |
| Acoustic Fatr (Hz) or ATrF | 0.42 | 1 | 0.59 | (-) |
| Acoustic Shimmer (apq5) | 0.43 | 1 | 0.60 | + |
| Acoustic DDP | 0.43 | 1 | 0.58 | + |
| Acoustic RAP (%) | 0.43 | 1 | 0.58 | + |
| Acoustic Shimmer (apq3) | 0.48 | 1 | 0.60 | + |
| Acoustic Shimmer (dda) | 0.48 | 1 | 0.60 | + |
| Acoustic Shim (%) | 0.49 | 1 | 0.61 | + |
| Acoustic F0 (Hz) | 0.52 | 1 | 0.58 | (-) |
| Acoustic F3 | 0.52 | 1 | 0.61 | + |
| Syllables per word min | 0.54 | 1 | 0.58 | + |
| Acoustic F5 | 0.54 | 1 | 0.61 | + |
| Acoustic PPQ (%) | 0.54 | 1 | 0.58 | + |
| Number of Adverbs | 0.55 | 1 | 0.59 | + |
| Demonstrative | 0.55 | 1 | 0.59 | (-) |
| Question Words | 0.56 | 1 | 0.60 | + |
| Acoustic VTI | 0.56 | 1 | 0.62 | (-) |
| Count of complete sentences | 0.57 | 1 | 0.59 | (-) |
| Acoustic SPI | 0.58 | 1 | 0.59 | + |
| Count of pronouns | 0.62 | 1 | 0.59 | + |
| Count of content units on LEFT side of picture | 0.62 | 1 | 0.61 | (-) |
| Acoustics voice breaks percentage | 0.62 | 1 | 0.60 | + |
| Acoustic ATRI (%) or ATrI | 0.62 | 1 | 0.58 | + |
| Definite Article | 0.63 | 1 | 0.60 | + |
| SD Number of Syllables / Word | 0.69 | 1 | 0.63 | (-) |
| Acoustic PFR | 0.69 | 1 | 0.61 | (-) |
| Acoustic Harmonicity_Stdev | 0.70 | 1 | 0.60 | (-) |
| Acoustic F1 | 0.70 | 1 | 0.61 | + |
| Acoustic ATrP | 0.71 | 1 | 0.58 | + |
| Acoustic Fhi (Hz) | 0.72 | 1 | 0.60 | (-) |
| Acoustic NVB (%) | 0.74 | 1 | 0.60 | + |
| Acoustics standard deviation of F0 | 0.76 | 1 | 0.59 | + |
| Possessive Determiners | 0.76 | 1 | 0.60 | (-) |
| Acoustic F4 | 0.79 | 1 | 0.61 | + |
| Acoustic F2 | 0.81 | 1 | 0.61 | + |
| Acoustic FTRI (%) or FTrI | 0.81 | 1 | 0.60 | (-) |
| Count of all function words | 0.83 | 1 | 0.60 | (-) |
| Count of difference words: other, another | 0.84 | 1 | 0.60 | (-) |
| Acoustic FTrP | 0.86 | 1 | 0.59 | + |
| Conjunction | 0.87 | 1 | 0.60 | (-) |
| Count of quantifiers | 0.87 | 1 | 0.62 | + |
| Acoustic Flo (Hz) | 0.87 | 1 | 0.61 | + |
| Syllables per word max | 0.89 | 1 | 0.62 | (-) |
| Count of distributive determiners | 0.90 | 1 | 0.62 | (-) |
| Numbers | 0.90 | 1 | 0.61 | + |
| Auxiliary Verbs | 0.92 | 1 | 0.59 | + |
| Qualifiers | 0.93 | 1 | 0.62 | + |
| Acoustic F0spread16to50 | 0.95 | 1 | 0.60 | (-) |
| Number of Verbs | 0.97 | 1 | 0.59 | (-) |
| Pro-sentences | 1.00 | 1 | 0.59 | (-) |
| Count of pre-determiners | 1.00 | 1 | 0.59 | (-) |

**HC** = healthy control. **aMCI** = amnestic mild cognitive impairment. **naMCI** = nonamnestic mild cognitive impairment. **FDR** = Benjamini Yakutieli false discovery rate. **AUROC** = area under the receiver operator curve.


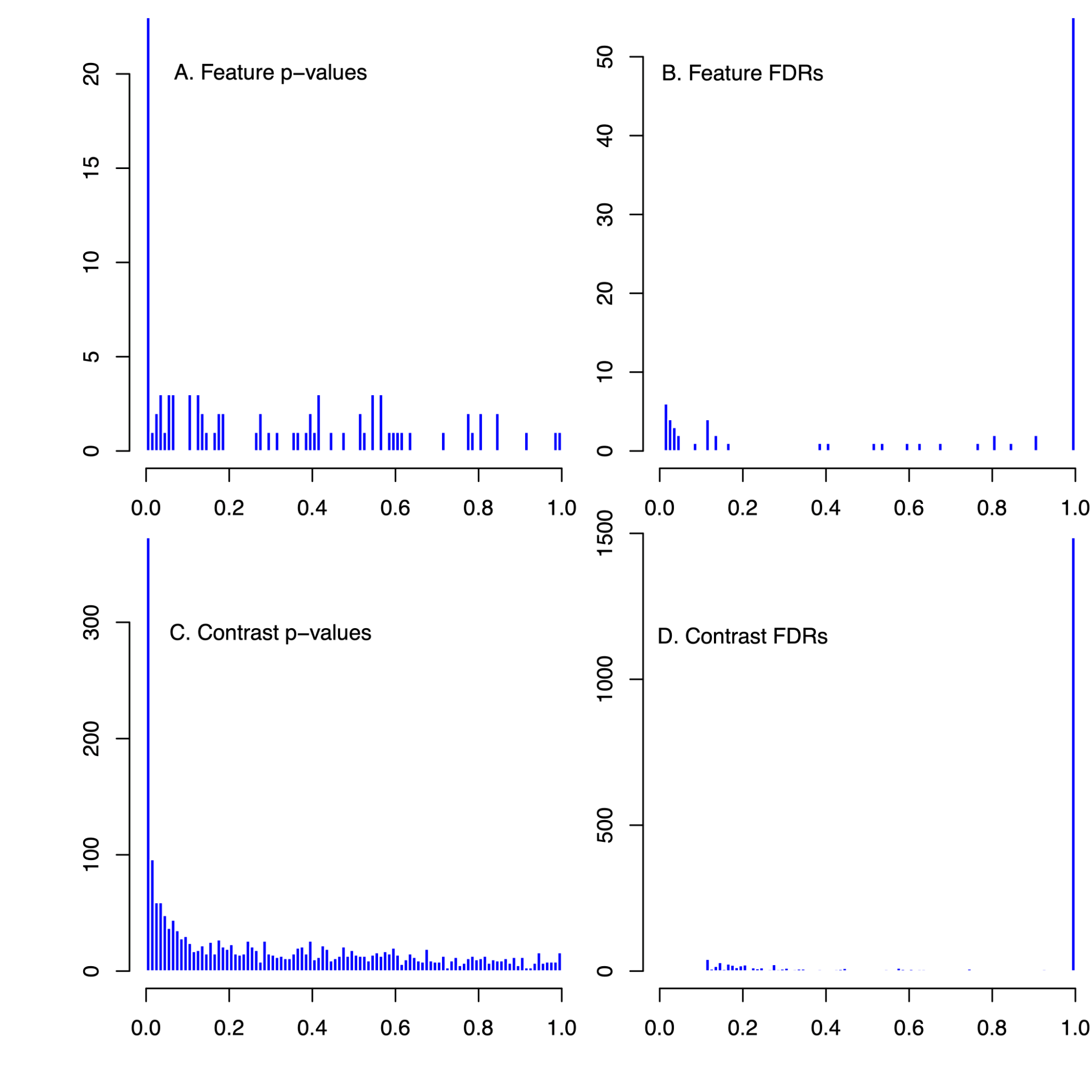


### Figure A. Association of Miro Health features and contrasts with aMCI vs HC

For 91 speech and language features based on analysis of transcripts of study participants’ responses in the Picture Description task or on acoustic analysis of their recorded speech. p-values were calculated for association with either aMCI or HC cohort membership. P-values were calculated for logistic regression models to predict cohort using quantile normalized speech or language features with adjustment for age and gender. The p-values were converted to false discovery rates by the Benjamini-Yekutieli method for dependent tests. P-values and FDRs were also calculated for 2007 contrasts by the same methods, where contrasts are new features defined as the difference between a pair of the features considered earlier. Contrasts were only considered between pairs of transcript-based language features or between pairs of acoustic speech features.

aMCI: Amnestic mild cognitive impairment. HC: healthy control. FDR: False discovery rate

1. P-values for 91 speech and language features.
2. FDRs for 91 speech and language features.
3. P-values for 2007 contrasts between pairs of quantile normalized speech and language features.
4. FDRs for 2007 contrasts between pairs of quantile normalized speech and language features.


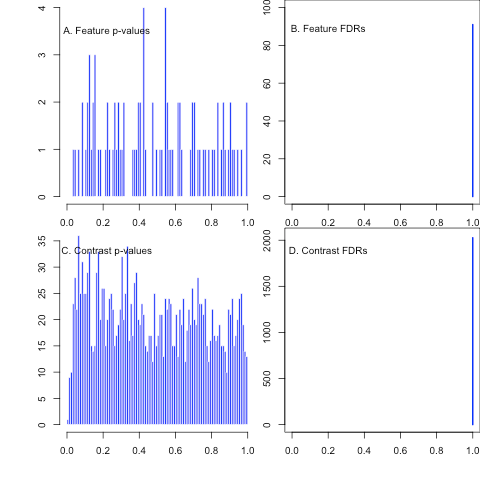


### Figure B. Association of Miro Health features and contrasts with naMCI vs HC

###

For 91 speech and language features based on analysis of transcripts of study participants’ responses in the Picture Description task or on acoustic analysis of their recorded speech p-values were calculated for association with either naMCI or HC cohort membership. P-values were calculated for logistic regression models to predict cohort using quantile normalized speech or language features with adjustment for age and gender. The p-values were converted to false discovery rates by the Benjamini-Yekutieli method for dependent tests. P-values and FDRs were also calculated for 2007 contrasts by the same methods, where contrasts are new features defined as the difference between a pair of the speech or language features considered earlier. Contrasts were only considered between pairs of transcript-based language features or between pairs of acoustic speech features.

naMCI: non-amnestic mild cognitive impairment. HC: healthy control. FDR: False discovery rate

1. P-values for 91 speech and language features.
2. FDRs for 91 speech and language features.
3. P-values for 2007 contrasts between pairs of quantile normalized speech and language features.
4. FDRs for 2007 contrasts between pairs of quantile normalized speech and language features.

# D. Contrasts

### Table F. Association with membership in the aMCI or HC cohorts of contrasts (A-B) of normalized values of two speech or language features A and B.

| **Speech or Language**  **Feature A** | **Speech or Language**  **Feature B** | **p-value** | **FDR** | **AUROC** | **Effect**  **Direction** |
| --- | --- | --- | --- | --- | --- |
| Count of modals | Count of prepositions | 4.9E-05 | 0.114 | 0.86 | 1 |
| Count of modals | Count of RELATED content units | 7.5E-05 | 0.114 | 0.89 | 1 |
| Pro-sentences | Count of prepositions | 8.5E-05 | 0.114 | 0.84 | 1 |
| Count of modals | Count of indefinite articles | 8.6E-05 | 0.114 | 0.86 | 1 |
| Count of modals | Count of nouns | 1.1E-04 | 0.114 | 0.88 | 1 |
| Pro-sentences | Count of RELATED content units | 1.1E-04 | 0.114 | 0.85 | 1 |
| Count of modals | Both Sides Total Content Units Unique | 1.1E-04 | 0.114 | 0.85 | 1 |
| Count of modals | Count of words | 1.1E-04 | 0.114 | 0.84 | 1 |
| Syllables per word min | Count of DISTINCT RELATED content units | 1.1E-04 | 0.114 | 0.83 | 1 |
| Count of modals | Count of content units on RIGHT side of picture | 1.2E-04 | 0.114 | 0.84 | 1 |
| Count of modals | Count of Syllables | 1.2E-04 | 0.114 | 0.84 | 1 |
| Syllables per word min | Count of RELATED content units | 1.2E-04 | 0.114 | 0.84 | 1 |
| Pro-sentences | Count of DISTINCT RELATED content units | 1.3E-04 | 0.114 | 0.84 | 1 |
| Pro-sentences | Count of Syllables | 1.3E-04 | 0.114 | 0.81 | 1 |
| Count of modals | Count of ALL content units | 1.3E-04 | 0.114 | 0.84 | 1 |
| Pro-sentences | Count of nouns | 1.3E-04 | 0.114 | 0.86 | 1 |
| Count of modals | Both Sides Total Content Units | 1.3E-04 | 0.114 | 0.83 | 1 |
| Count of modals | Count of ALL DISTINCT content units | 1.4E-04 | 0.114 | 0.83 | 1 |
| Pro-sentences | Count of ALL content units | 1.4E-04 | 0.114 | 0.82 | 1 |
| Count of modals | Count of DISTINCT RELATED content units | 1.5E-04 | 0.114 | 0.89 | 1 |
| Pro-sentences | Count of words | 1.5E-04 | 0.114 | 0.81 | 1 |
| Pro-sentences | Both Sides Total Content Units Unique | 1.6E-04 | 0.114 | 0.82 | 1 |
| Count of indefinite articles | Syllables per word min | 1.7E-04 | 0.114 | 0.83 | -1 |
| Count of modals | Count of phrases | 1.8E-04 | 0.114 | 0.81 | 1 |
| Count of pre-determiners | Count of DISTINCT RELATED content units | 1.8E-04 | 0.114 | 0.81 | 1 |
| Pro-sentences | Count of content units on RIGHT side of picture | 1.8E-04 | 0.114 | 0.81 | 1 |
| Count of prepositions | Syllables per word min | 2.1E-04 | 0.115 | 0.81 | -1 |
| Count of pre-determiners | Count of RELATED content units | 2.2E-04 | 0.115 | 0.81 | 1 |
| Pro-sentences | Count of phrases | 2.2E-04 | 0.115 | 0.8 | 1 |
| Count of pre-determiners | Count of prepositions | 2.3E-04 | 0.115 | 0.79 | 1 |
| Syllables per word min | Count of nouns | 2.3E-04 | 0.115 | 0.83 | 1 |
| Count of modals | Count of all function words | 2.4E-04 | 0.115 | 0.78 | 1 |
| Count of difference words: other,another | Count of prepositions | 2.5E-04 | 0.115 | 0.78 | 1 |
| Pro-sentences | Both Sides Total Content Units | 2.5E-04 | 0.115 | 0.8 | 1 |
| Syllables per word min | Both Sides Total Content Units Unique | 2.5E-04 | 0.115 | 0.81 | 1 |
| Count of difference words: other,another | Count of RELATED content units | 2.6E-04 | 0.115 | 0.81 | 1 |
| Pro-sentences | Count of ALL DISTINCT content units | 2.6E-04 | 0.115 | 0.8 | 1 |
| Pro-sentences | Count of indefinite articles | 2.6E-04 | 0.115 | 0.82 | 1 |
| Count of difference words: other,another | Count of nouns | 2.7E-04 | 0.115 | 0.82 | 1 |
| Count of modals | Count of DISTINCT content units on RIGHT side of picture | 2.8E-04 | 0.115 | 0.79 | 1 |
| Count of distributive determiners | Count of prepositions | 2.9E-04 | 0.118 | 0.77 | 1 |
| Count of difference words: other,another | Count of DISTINCT RELATED content units | 3.3E-04 | 0.123 | 0.79 | 1 |
| Count of quantifiers | Count of RELATED content units | 3.3E-04 | 0.123 | 0.8 | 1 |
| Syllables per word min | Both Sides Total Content Units | 3.3E-04 | 0.123 | 0.79 | 1 |
| Acoustics standard deviation of F0 | Acoustic NVB (%) | 3.4E-04 | 0.123 | 0.78 | 1 |
| Count of pre-determiners | Count of indefinite articles | 3.4E-04 | 0.123 | 0.79 | 1 |
| Syllables per word min | Count of ALL content units | 3.7E-04 | 0.129 | 0.77 | 1 |
| Count of pre-determiners | Count of nouns | 3.8E-04 | 0.129 | 0.8 | 1 |
| Count of prepositions | Count of pronouns | 3.8E-04 | 0.129 | 0.82 | -1 |
| Count of distributive determiners | Count of DISTINCT RELATED content units | 4.0E-04 | 0.132 | 0.78 | 1 |
| Syllables per word min | Count of content units on RIGHT side of picture | 4.1E-04 | 0.132 | 0.77 | 1 |
| Pro-sentences | Count of all function words | 4.3E-04 | 0.132 | 0.77 | 1 |
| Count of distributive determiners | Count of RELATED content units | 4.5E-04 | 0.132 | 0.8 | 1 |
| Count of modals | Count of DISTINCT content units on Left side of picture | 4.5E-04 | 0.132 | 0.75 | 1 |
| Count of indefinite articles | Count of utterances | 4.6E-04 | 0.132 | 0.75 | -1 |
| Count of prepositions | Count of utterances | 4.6E-04 | 0.132 | 0.77 | -1 |
| Count of pre-determiners | Both Sides Total Content Units Unique | 4.8E-04 | 0.132 | 0.76 | 1 |
| Count of utterances | Count of RELATED content units | 4.8E-04 | 0.132 | 0.76 | 1 |
| Count of distributive determiners | Count of nouns | 4.9E-04 | 0.132 | 0.81 | 1 |
| Count of pre-determiners | Count of content units on RIGHT side of picture | 4.9E-04 | 0.132 | 0.76 | 1 |
| Count of modals | Count of sentences | 5.0E-04 | 0.132 | 0.76 | 1 |

# E. Distributions of selected acoustic features across diagnostic cohort (aMCI, naMCI, HC) and gender


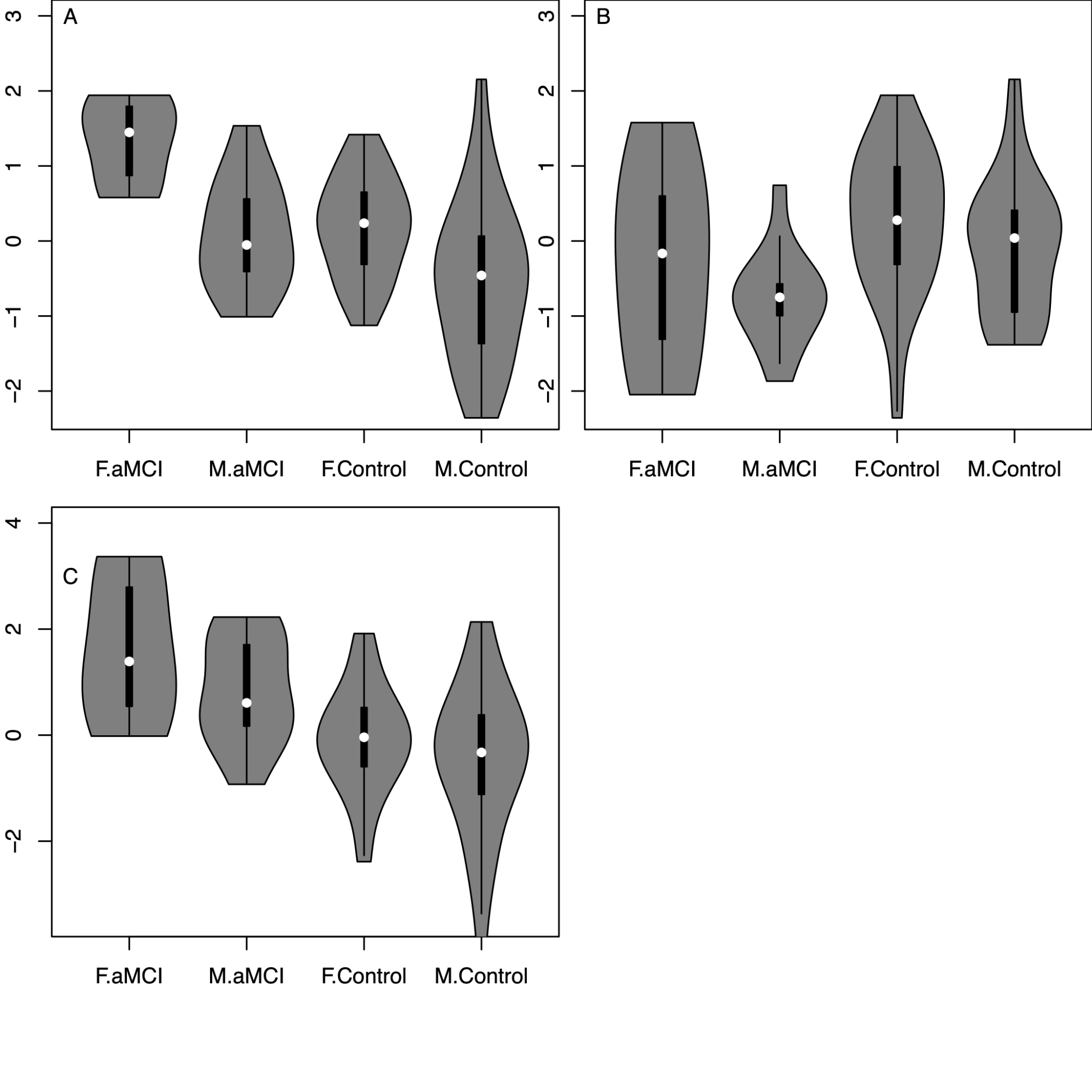


### Figure C.

###

Violin plots for quantile normalized acoustic speech features and their contrast. The plots show the differences in the distributions of these quantities across study cohorts (aMCI or healthy control) and gender. aMCI: amnestic mild cognitive impairment. F: female. M: male. F0 speech fundamental frequency.

A: Standard deviation of fundamental frequency F0.

B: Speech breaks.

C: Contrast (Standard deviation of fundamental frequency F0) - (Speech breaks).

# F. Classification Performance

### Table G. HC vs aMCI classification performance measures for Miro Health picture description features

| **Variable** | **AUROC** | **Accuracy** | **Sensitivity** | **Specificity** | **Precision** | **F1** |
| --- | --- | --- | --- | --- | --- | --- |
| Count of RELATED content units | 0.83 | 0.83 | 0.66 | 0.88 | 0.61 | 0.63 |
| Count of DISTINCT RELATED content units | 0.82 | 0.81 | 0.55 | 0.89 | 0.61 | 0.57 |
| Count of indefinite articles | 0.82 | 0.82 | 0.68 | 0.86 | 0.59 | 0.63 |
| Count of nouns | 0.81 | 0.84 | 0.68 | 0.89 | 0.63 | 0.65 |
| Both Sides Total Content Units Unique | 0.80 | 0.79 | 0.49 | 0.87 | 0.53 | 0.51 |
| Count of prepositions | 0.79 | 0.83 | 0.58 | 0.91 | 0.66 | 0.61 |
| Both Sides Total Content Units | 0.78 | 0.78 | 0.42 | 0.89 | 0.54 | 0.46 |
| Count of ALL content units | 0.76 | 0.79 | 0.51 | 0.87 | 0.54 | 0.52 |
| Count of content units on RIGHT side of picture | 0.75 | 0.77 | 0.39 | 0.88 | 0.49 | 0.43 |
| Count of Syllables | 0.74 | 0.80 | 0.47 | 0.90 | 0.57 | 0.51 |
| Count of ALL DISTINCT content units | 0.73 | 0.79 | 0.45 | 0.88 | 0.54 | 0.49 |
| Count of words | 0.73 | 0.80 | 0.47 | 0.89 | 0.56 | 0.51 |
| Count of DISTINCT content units on RIGHT side of picture | 0.71 | 0.75 | 0.32 | 0.87 | 0.42 | 0.36 |
| Count of phrases | 0.71 | 0.79 | 0.43 | 0.89 | 0.54 | 0.47 |
| Acoustics standard deviation of F0 | 0.71 | 0.76 | 0.36 | 0.88 | 0.46 | 0.40 |
| Count of sentences | 0.69 | 0.78 | 0.36 | 0.90 | 0.52 | 0.42 |
| Count of all function words | 0.67 | 0.77 | 0.36 | 0.89 | 0.50 | 0.41 |
| Acoustics voice breaks percentage | 0.67 | 0.74 | 0.23 | 0.88 | 0.36 | 0.27 |
| Count of complete sentences | 0.66 | 0.77 | 0.43 | 0.87 | 0.49 | 0.45 |
| Count of content units on LEFT side of picture | 0.66 | 0.72 | 0.10 | 0.90 | 0.22 | 0.13 |
| Count of DISTINCT content units on Left side of picture | 0.65 | 0.74 | 0.09 | 0.93 | 0.34 | 0.13 |
| Syllables: Utterances Ratio | 0.64 | 0.73 | 0.07 | 0.92 | 0.21 | 0.10 |
| Numbers | 0.61 | 0.73 | 0.26 | 0.87 | 0.35 | 0.29 |
| Acoustic NVB (%) | 0.61 | 0.75 | 0.13 | 0.93 | 0.44 | 0.19 |
| Acoustic F0spread50to84 | 0.61 | 0.75 | 0.27 | 0.89 | 0.41 | 0.31 |
| Number of Verbs | 0.60 | 0.74 | 0.25 | 0.88 | 0.37 | 0.30 |
| Acoustic RAP (%) | 0.59 | 0.76 | 0.26 | 0.91 | 0.54 | 0.32 |
| Acoustic DDP | 0.59 | 0.77 | 0.26 | 0.92 | 0.60 | 0.33 |
| Total Function Words / Content Units | 0.59 | 0.77 | 0.28 | 0.91 | 0.51 | 0.35 |
| Acoustic DUV | 0.59 | 0.73 | 0.19 | 0.89 | 0.32 | 0.24 |
| Total Function Words / Total Words | 0.59 | 0.78 | 0.26 | 0.93 | 0.58 | 0.34 |
| Acoustic F3 | 0.58 | 0.73 | 0.28 | 0.86 | 0.37 | 0.31 |
| Acoustic PPQ (%) | 0.58 | 0.77 | 0.26 | 0.91 | 0.52 | 0.32 |
| Acoustic Jita (s) | 0.57 | 0.76 | 0.27 | 0.90 | 0.51 | 0.33 |
| Acoustic Tsam (sec) | 0.57 | 0.74 | 0.23 | 0.89 | 0.36 | 0.28 |
| Variability (vector distance) | 0.57 | 0.77 | 0.26 | 0.92 | 0.58 | 0.34 |
| Demonstrative | 0.57 | 0.77 | 0.16 | 0.94 | 0.54 | 0.23 |
| Total Content Units / Total Words | 0.57 | 0.78 | 0.26 | 0.93 | 0.66 | 0.34 |
| Acoustic Harmonicity_Stdev | 0.57 | 0.74 | 0.26 | 0.88 | 0.39 | 0.31 |
| Acoustic FTrP | 0.57 | 0.78 | 0.26 | 0.93 | 0.65 | 0.35 |
| Count of distributive determiners | 0.57 | 0.78 | 0.29 | 0.93 | 0.61 | 0.37 |
| Acoustic Shimmer (apq3) | 0.57 | 0.76 | 0.26 | 0.91 | 0.48 | 0.32 |
| Acoustic Harmonicity_90p | 0.57 | 0.77 | 0.26 | 0.92 | 0.53 | 0.33 |
| Conjunction | 0.57 | 0.73 | 0.18 | 0.88 | 0.31 | 0.23 |
| Acoustic Shimmer (dda) | 0.56 | 0.76 | 0.27 | 0.90 | 0.48 | 0.33 |
| Acoustic FTRI (%) or FTrI | 0.56 | 0.77 | 0.29 | 0.92 | 0.58 | 0.37 |
| Acoustic F0 (Hz) | 0.56 | 0.75 | 0.24 | 0.90 | 0.43 | 0.30 |
| Acoustic Shimmer (apq5) | 0.56 | 0.76 | 0.24 | 0.91 | 0.47 | 0.31 |
| Count of utterances | 0.56 | 0.74 | 0.27 | 0.87 | 0.39 | 0.32 |
| Acoustic ATRI (%) or ATrI | 0.56 | 0.74 | 0.15 | 0.91 | 0.35 | 0.20 |
| Acoustic Mean_absolute_slope | 0.56 | 0.76 | 0.27 | 0.90 | 0.47 | 0.33 |
| Acoustic VTI | 0.56 | 0.77 | 0.26 | 0.91 | 0.50 | 0.33 |
| Definite Article | 0.56 | 0.73 | 0.09 | 0.91 | 0.26 | 0.12 |
| Acoustic PFR | 0.56 | 0.72 | 0.16 | 0.88 | 0.27 | 0.20 |
| Acoustic Fhi (Hz) | 0.56 | 0.73 | 0.17 | 0.89 | 0.30 | 0.20 |
| Acoustic Shdb (dB) | 0.56 | 0.76 | 0.22 | 0.91 | 0.53 | 0.28 |
| Acoustic F0spread16to50 | 0.56 | 0.75 | 0.24 | 0.90 | 0.43 | 0.30 |
| Acoustic Jitt (%) | 0.55 | 0.76 | 0.21 | 0.92 | 0.53 | 0.27 |
| Acoustic Flo (Hz) | 0.55 | 0.77 | 0.23 | 0.93 | 0.62 | 0.31 |
| Acoustic Fftr (Hz) or FTrF | 0.55 | 0.77 | 0.18 | 0.94 | 0.55 | 0.26 |
| Number of Adverbs | 0.55 | 0.73 | 0.19 | 0.88 | 0.34 | 0.24 |
| Acoustic Harmonicity_10p | 0.55 | 0.77 | 0.14 | 0.95 | 0.50 | 0.22 |
| Possessive Determiners | 0.55 | 0.77 | 0.19 | 0.94 | 0.56 | 0.28 |
| Acoustic Shim (%) | 0.55 | 0.75 | 0.21 | 0.90 | 0.44 | 0.27 |
| Count of modals | 0.55 | 0.75 | 0.11 | 0.93 | 0.36 | 0.15 |
| SD Number of Syllables / Word | 0.55 | 0.79 | 0.29 | 0.93 | 0.64 | 0.38 |
| Particles | 0.55 | 0.76 | 0.20 | 0.92 | 0.47 | 0.27 |
| Pro-sentences | 0.55 | 0.77 | 0.21 | 0.93 | 0.53 | 0.28 |
| Acoustic SPI | 0.55 | 0.75 | 0.24 | 0.90 | 0.49 | 0.30 |
| Acoustic ATrP | 0.55 | 0.76 | 0.18 | 0.93 | 0.45 | 0.25 |
| Acoustic APQ (%) | 0.55 | 0.76 | 0.19 | 0.92 | 0.49 | 0.26 |
| Acoustic Fatr (Hz) or ATrF | 0.55 | 0.74 | 0.03 | 0.95 | 0.10 | 0.04 |
| Acoustic Harmonicity_50p | 0.55 | 0.75 | 0.23 | 0.90 | 0.49 | 0.29 |
| Count of pre-determiners | 0.55 | 0.76 | 0.26 | 0.90 | 0.44 | 0.32 |
| Syllables per word max | 0.54 | 0.75 | 0.24 | 0.90 | 0.45 | 0.30 |
| Syllables: Word Ratio (mean) | 0.54 | 0.77 | 0.25 | 0.91 | 0.50 | 0.32 |
| Auxiliary Verbs | 0.54 | 0.72 | 0.17 | 0.89 | 0.30 | 0.21 |
| Acoustic NHR | 0.54 | 0.76 | 0.18 | 0.92 | 0.48 | 0.25 |
| Acoustic F4 | 0.54 | 0.72 | 0.21 | 0.87 | 0.31 | 0.25 |
| Acoustic F5 | 0.54 | 0.74 | 0.11 | 0.92 | 0.33 | 0.15 |
| Qualifiers | 0.54 | 0.76 | 0.25 | 0.91 | 0.53 | 0.31 |
| Acoustic Harmonicity_Average | 0.54 | 0.76 | 0.23 | 0.91 | 0.46 | 0.30 |
| Syllables per word min | 0.54 | 0.78 | 0.23 | 0.94 | 0.60 | 0.31 |
| Count of pronouns | 0.54 | 0.76 | 0.22 | 0.92 | 0.50 | 0.28 |
| Acoustic NUV | 0.54 | 0.79 | 0.21 | 0.96 | 0.70 | 0.30 |
| Acoustic F2 | 0.54 | 0.74 | 0.19 | 0.89 | 0.33 | 0.24 |
| Acoustic F1 | 0.54 | 0.72 | 0.14 | 0.89 | 0.26 | 0.18 |
| Count of difference words: other, another | 0.53 | 0.78 | 0.26 | 0.93 | 0.62 | 0.34 |
| Count of quantifiers | 0.53 | 0.74 | 0.23 | 0.89 | 0.42 | 0.28 |
| Acoustic DVB (%) | 0.53 | 0.71 | 0.10 | 0.89 | 0.21 | 0.13 |
| Question Words | 0.53 | 0.78 | 0.18 | 0.95 | 0.64 | 0.27 |

**HC** = healthy control. **aMCI** = amnestic mild cognitive impairment.  **AUROC** = area under the receiver operator curve.

### Table H. HC vs naMCI classification performance measures for Miro Health picture description features

| **Variable** | **AUROC** | **Accuracy** | **Sensitivity** | **Specificity** | **Precision** | **F1** |
| --- | --- | --- | --- | --- | --- | --- |
| SD Number of Syllables / Word | 0.63 | 0.79 | 0.35 | 0.91 | 0.57 | 0.41 |
| Count of distributive determiners | 0.62 | 0.78 | 0.30 | 0.91 | 0.54 | 0.36 |
| Qualifiers | 0.62 | 0.79 | 0.27 | 0.93 | 0.56 | 0.35 |
| Syllables per word max | 0.62 | 0.79 | 0.33 | 0.91 | 0.55 | 0.40 |
| Acoustic VTI | 0.62 | 0.79 | 0.26 | 0.93 | 0.57 | 0.34 |
| Count of quantifiers | 0.62 | 0.80 | 0.31 | 0.93 | 0.60 | 0.39 |
| Acoustic F5 | 0.61 | 0.76 | 0.08 | 0.95 | 0.18 | 0.10 |
| Acoustic F4 | 0.61 | 0.75 | 0.11 | 0.92 | 0.18 | 0.13 |
| Acoustic F1 | 0.61 | 0.74 | 0.08 | 0.92 | 0.14 | 0.10 |
| Acoustic F2 | 0.61 | 0.76 | 0.07 | 0.94 | 0.16 | 0.10 |
| Count of content units on LEFT side of picture | 0.61 | 0.79 | 0.30 | 0.92 | 0.55 | 0.37 |
| Acoustic Flo (Hz) | 0.61 | 0.78 | 0.30 | 0.92 | 0.55 | 0.37 |
| Numbers | 0.61 | 0.76 | 0.11 | 0.94 | 0.28 | 0.14 |
| Acoustic Shim (%) | 0.61 | 0.80 | 0.28 | 0.94 | 0.66 | 0.36 |
| Acoustic PFR | 0.61 | 0.76 | 0.09 | 0.94 | 0.19 | 0.12 |
| Acoustic F3 | 0.61 | 0.76 | 0.07 | 0.94 | 0.17 | 0.10 |
| Conjunction | 0.60 | 0.76 | 0.11 | 0.93 | 0.19 | 0.13 |
| Definite Article | 0.60 | 0.79 | 0.30 | 0.92 | 0.56 | 0.38 |
| Possessive Determiners | 0.60 | 0.79 | 0.30 | 0.92 | 0.52 | 0.37 |
| Acoustic Shimmer (dda) | 0.60 | 0.80 | 0.23 | 0.95 | 0.67 | 0.33 |
| Acoustic Shimmer (apq5) | 0.60 | 0.77 | 0.26 | 0.91 | 0.43 | 0.32 |
| Count of all function words | 0.60 | 0.77 | 0.11 | 0.94 | 0.23 | 0.15 |
| Acoustic Fhi (Hz) | 0.60 | 0.76 | 0.10 | 0.93 | 0.19 | 0.13 |
| Acoustic Shdb (dB) | 0.60 | 0.77 | 0.23 | 0.92 | 0.48 | 0.30 |
| Acoustic NVB (%) | 0.60 | 0.78 | 0.33 | 0.91 | 0.50 | 0.39 |
| Count of difference words: other, another | 0.60 | 0.77 | 0.28 | 0.90 | 0.45 | 0.34 |
| Acoustic Harmonicity_Stdev | 0.60 | 0.77 | 0.09 | 0.95 | 0.25 | 0.14 |
| Acoustic F0spread16to50 | 0.60 | 0.80 | 0.33 | 0.92 | 0.55 | 0.41 |
| Acoustic Shimmer (apq3) | 0.60 | 0.79 | 0.24 | 0.94 | 0.58 | 0.33 |
| Acoustic FTRI (%) or FTrI | 0.60 | 0.80 | 0.32 | 0.93 | 0.57 | 0.40 |
| Count of DISTINCT content units on Left side of picture | 0.60 | 0.77 | 0.18 | 0.93 | 0.50 | 0.25 |
| Both Sides Total Content Units | 0.60 | 0.76 | 0.11 | 0.93 | 0.21 | 0.13 |
| Acoustics voice breaks percentage | 0.60 | 0.75 | 0.08 | 0.92 | 0.16 | 0.10 |
| Particles | 0.60 | 0.76 | 0.29 | 0.88 | 0.40 | 0.33 |
| Question Words | 0.60 | 0.80 | 0.33 | 0.93 | 0.56 | 0.40 |
| Acoustics standard deviation of F0 | 0.59 | 0.75 | 0.13 | 0.91 | 0.21 | 0.16 |
| Acoustic SPI | 0.59 | 0.77 | 0.29 | 0.91 | 0.47 | 0.34 |
| Both Sides Total Content Units Unique | 0.59 | 0.76 | 0.12 | 0.93 | 0.17 | 0.14 |
| Demonstrative | 0.59 | 0.77 | 0.26 | 0.90 | 0.43 | 0.32 |
| Auxiliary Verbs | 0.59 | 0.76 | 0.12 | 0.93 | 0.24 | 0.16 |
| Number of Verbs | 0.59 | 0.77 | 0.10 | 0.95 | 0.24 | 0.14 |
| Acoustic Harmonicity_90p | 0.59 | 0.79 | 0.24 | 0.94 | 0.50 | 0.29 |
| Acoustic F0spread50to84 | 0.59 | 0.75 | 0.11 | 0.92 | 0.19 | 0.14 |
| Count of complete sentences | 0.59 | 0.77 | 0.10 | 0.94 | 0.23 | 0.13 |
| Pro-sentences | 0.59 | 0.77 | 0.24 | 0.91 | 0.43 | 0.30 |
| Number of Adverbs | 0.59 | 0.76 | 0.15 | 0.92 | 0.23 | 0.18 |
| Count of pre-determiners | 0.59 | 0.78 | 0.29 | 0.91 | 0.50 | 0.36 |
| Count of words | 0.59 | 0.76 | 0.08 | 0.94 | 0.16 | 0.10 |
| Count of sentences | 0.59 | 0.75 | 0.12 | 0.92 | 0.20 | 0.15 |
| Acoustic NUV | 0.59 | 0.78 | 0.18 | 0.94 | 0.51 | 0.25 |
| Count of ALL DISTINCT content units | 0.59 | 0.76 | 0.10 | 0.93 | 0.17 | 0.12 |
| Acoustic FTrP | 0.59 | 0.77 | 0.28 | 0.90 | 0.44 | 0.33 |
| Acoustic APQ (%) | 0.59 | 0.77 | 0.27 | 0.91 | 0.47 | 0.33 |
| Acoustic Fatr (Hz) or ATrF | 0.59 | 0.76 | 0.12 | 0.93 | 0.21 | 0.15 |
| Acoustic Fftr (Hz) or FTrF | 0.59 | 0.76 | 0.17 | 0.92 | 0.34 | 0.22 |
| Count of pronouns | 0.59 | 0.79 | 0.32 | 0.91 | 0.50 | 0.38 |
| Total Content Units / Total Words | 0.59 | 0.77 | 0.24 | 0.91 | 0.40 | 0.29 |
| Acoustic Harmonicity_Average | 0.59 | 0.79 | 0.24 | 0.93 | 0.59 | 0.32 |
| Acoustic NHR | 0.59 | 0.77 | 0.22 | 0.92 | 0.40 | 0.27 |
| Count of Syllables | 0.59 | 0.75 | 0.07 | 0.93 | 0.12 | 0.08 |
| Total Function Words / Content Units | 0.58 | 0.76 | 0.22 | 0.91 | 0.39 | 0.27 |
| Syllables per word min | 0.58 | 0.77 | 0.28 | 0.90 | 0.46 | 0.34 |
| Count of phrases | 0.58 | 0.75 | 0.10 | 0.93 | 0.15 | 0.12 |
| Acoustic DDP | 0.58 | 0.77 | 0.24 | 0.91 | 0.43 | 0.29 |
| Acoustic ATrP | 0.58 | 0.78 | 0.33 | 0.91 | 0.49 | 0.39 |
| Acoustic F0 (Hz) | 0.58 | 0.77 | 0.25 | 0.91 | 0.42 | 0.30 |
| Acoustic RAP (%) | 0.58 | 0.77 | 0.24 | 0.91 | 0.42 | 0.28 |
| Count of ALL content units | 0.58 | 0.76 | 0.09 | 0.94 | 0.18 | 0.12 |
| Acoustic PPQ (%) | 0.58 | 0.78 | 0.25 | 0.92 | 0.50 | 0.30 |
| Acoustic Jitt (%) | 0.58 | 0.77 | 0.23 | 0.92 | 0.47 | 0.28 |
| Total Function Words / Total Words | 0.58 | 0.75 | 0.21 | 0.90 | 0.33 | 0.25 |
| Acoustic ATRI (%) or ATrI | 0.58 | 0.77 | 0.30 | 0.90 | 0.44 | 0.35 |
| Acoustic Harmonicity_50p | 0.58 | 0.79 | 0.25 | 0.93 | 0.59 | 0.32 |
| Acoustic Harmonicity_10p | 0.58 | 0.76 | 0.21 | 0.91 | 0.44 | 0.26 |
| Count of indefinite articles | 0.58 | 0.76 | 0.16 | 0.92 | 0.23 | 0.18 |
| Count of DISTINCT content units on RIGHT side of picture | 0.58 | 0.75 | 0.11 | 0.93 | 0.20 | 0.14 |
| Count of prepositions | 0.58 | 0.74 | 0.08 | 0.92 | 0.13 | 0.09 |
| Count of RELATED content units | 0.58 | 0.75 | 0.14 | 0.92 | 0.23 | 0.17 |
| Syllables: Word Ratio (mean) | 0.58 | 0.77 | 0.14 | 0.94 | 0.59 | 0.20 |
| Acoustic DUV | 0.57 | 0.75 | 0.25 | 0.89 | 0.37 | 0.29 |
| Count of modals | 0.57 | 0.77 | 0.14 | 0.94 | 0.29 | 0.18 |
| Count of nouns | 0.57 | 0.76 | 0.11 | 0.93 | 0.18 | 0.13 |
| Acoustic Tsam (sec) | 0.57 | 0.76 | 0.17 | 0.92 | 0.32 | 0.21 |
| Acoustic Jita (s) | 0.57 | 0.77 | 0.23 | 0.91 | 0.49 | 0.28 |
| Count of content units on RIGHT side of picture | 0.57 | 0.74 | 0.11 | 0.91 | 0.17 | 0.13 |
| Count of DISTINCT RELATED content units | 0.57 | 0.76 | 0.15 | 0.92 | 0.23 | 0.18 |
| Acoustic Mean_absolute_slope | 0.57 | 0.76 | 0.23 | 0.90 | 0.42 | 0.28 |
| Variability (vector distance) | 0.57 | 0.77 | 0.22 | 0.92 | 0.47 | 0.29 |
| Acoustic DVB (%) | 0.56 | 0.76 | 0.17 | 0.91 | 0.32 | 0.22 |
| Count of utterances | 0.55 | 0.77 | 0.16 | 0.93 | 0.44 | 0.23 |
| Syllables: Utterances Ratio | 0.55 | 0.74 | 0.13 | 0.91 | 0.31 | 0.18 |

**HC** = healthy control. **naMCI** = nonamnestic mild cognitive impairment. **AUROC** = area under the receiver operator curve.

### Table I. Penalized multinomial logistic regression risk scores

| **Comparison** | **AUROC** |
| --- | --- |
| HC vs aMCI | 0.86 |
| HC vs naMCI | 0.51 |
| aMCI vs naMC | 0.78 |

**HC** = healthy control. **aMCI** = amnestic mild cognitive impairment.  **naMCI** = nonamnestic mild cognitive impairment. **AUROC** = area under the receiver operator curve.

### Table J. Classification performance measures for risk scores based on Miro Health picture description and on the full Miro Health assessment battery

| Model | AUROC | Accuracy | Sensitivity | Specificity | Precision | F1 |
| --- | --- | --- | --- | --- | --- | --- |
| PD risk score: aMCI vs HC | 0.88 | 0.86 | 0.72 | 0.90 | 0.68 | 0.70 |
| PD risk score: naMCI vs HC | 0.61 | 0.75 | 0.27 | 0.87 | 0.33 | 0.30 |
| PD risk score: (aMCI+naMCI) vs HC | 0.74 | 0.75 | 0.52 | 0.87 | 0.68 | 0.59 |
| Miro risk score: aMCI vs HC | 0.97 | 0.92 | 0.81 | 0.95 | 0.85 | 0.83 |
| Miro risk score: naMCI vs HC | 0.80 | 0.84 | 0.35 | 0.97 | 0.75 | 0.48 |
| Miro risk score: (aMCI+naMCI) vs HC | 0.89 | 0.82 | 0.74 | 0.86 | 0.76 | 0.75 |

# G. Risk score models from penalized logistic regression

### Table K. Regression coefficients from fitted penalized logistic regression models

| **Variable** | **HC vs aMCI** | **HC vs naMCI** | **HC vs (aMCI + naMCI)** |
| --- | --- | --- | --- |
| Intercept | -2.18026789 | -1.44 | -0.80820533 |
| Age | 0.150318176 | 0.197 | 0.09445665 |
| Gender | 1.195928737 | 0.291 | 0.6629267 |
| Acoustics standard deviation of F0 | 0.510032835 | - | 0.03209346 |
| Acoustic Harmonicity_10p | -0.006527359 | - | -0.0224571 |
| Acoustic F0spread50to84 | 0.328595418 | - | 0.22038086 |
| Acoustic Mean_absolute_slope | 0.234558848 | - | 0.13233809 |
| Acoustic Tsam (sec) | -0.115551663 | - | -0.16544995 |
| Count of content units on RIGHT side of picture | -0.290505423 | - | -0.19978974 |
| Count of RELATED content units | -0.183735107 | - | -0.14113808 |
| Count of DISTINCT RELATED content units | -0.251950549 | - | -0.0605782 |
| Count of indefinite articles | -0.772157529 | - | -0.37207445 |
| Count of modals | 0.77024371 | - | 0.30695817 |
| Count of prepositions | -0.450474329 | - | -0.23970264 |
| Acoustic NUV | - | - | -0.07956332 |
| Acoustics voice breaks percentage | - | - | 0.09698971 |
| Count of DISTINCT content units on RIGHT side of picture | - | - | -0.13776549 |
| Count of sentences | - | - | -0.02249432 |
| Syllables: Utterances Ratio | - | - | -0.07632111 |
| Syllables: Word Ratio (mean) | - | - | -0.04670185 |
| Variability (vector distance) | - | - | 0.02244452 |
| Acoustic Fhi (Hz) | 0.119898487 | - | - |
| Acoustic Flo (Hz) | -0.179366685 | - | - |
| Acoustic Harmonicity_Stdev | 0.150027726 | - | - |
| Both Sides Total Content Units Unique | -0.272506597 | - | - |
| Count of complete sentences | -0.018671628 | - | - |
| Count of nouns | -0.090591489 | - | - |
| Count of pronouns | 0.102796369 | - | - |

**HC** = healthy control. **aMCI** = amnestic mild cognitive impairment.  **naMCI** = nonamnestic mild cognitive impairment.

# H. Miro Health Picture Description feature names and definitions

### Table L. Miro Health Picture Description feature names and definitions

| **Variable** | **Definition** |
| --- | --- |
| Acoustic ATRI (%) or ATrI | Amplitude tremor intensity index shows (in percent) the ratio of the amplitude of the most intensive low-frequency amplitude-modulating component (amplitude tremor) to the total amplitude of the analyzed voice signal |
| Acoustic ATrP | Amplitude tremor power index |
| Acoustic DUV | The fraction of pitch frames that are analyzed as unvoiced |
| Acoustic DVB (%) | Degree of voice breaks shows the ratio of the total length of areas representing voice breaks to the time of the complete voice sample |
| Acoustic Fhi (Hz) | Highest fundamental frequency in the vocalization |
| Acoustic F0 (Hz) | Average fundamental frequency for the vocalization |
| Acoustic Flo (Hz) | Lowest fundamental frequency in the vocalization |
| Acoustics standard deviation of F0 | Standard deviation of the fundamental frequency in the vocalization |
| Acoustic Fatr (Hz) or ATrF | The frequency of the most intensive low-frequency amplitude-modulating component |
| Acoustic Fftr (Hz) or FTrF | The frequency of the most intensive low-frequency F0-modulating component |
| Acoustic F1 | Frequencies of the first five formants (F1‑F5) of selected Sound |
| Acoustic F2 | Frequencies of the first five formants (F1‑F5) of selected Sound |
| Acoustic F3 | Frequencies of the first five formants (F1‑F5) of selected Sound |
| Acoustic F4 | Frequencies of the first five formants (F1‑F5) of selected Sound |
| Acoustic F5 | Frequencies of the first five formants (F1‑F5) of selected Sound |
| Acoustic FTRI (%) or FTrI | F0-tremor intensity index shows (in percent) the ratio of the frequency magnitude of the most intensive low-frequency modulating component (F0-tremor) to the total frequency magnitude of the analyzed voice signal |
| Acoustic FTrP | Frequency tremor power index |
| Acoustic Harmonicity_10p | Periodicity-to-noise 10% of sounding frames in dB |
| Acoustic Harmonicity_50p | Periodicity-to-noise 50% of sounding frames in dB |
| Acoustic Harmonicity_90p | Periodicity-to-noise 90% of sounding frames in dB |
| Acoustic Harmonicity_Average | Average Periodicity-to-noise of sounding frames in dB |
| Acoustic Harmonicity_Stdev | Standard Deviation of Periodicity-to-noise of sounding frames in dB |
| Acoustic Jita (s) | Absolute jitter gives an evaluation in microseconds of the period-to-period variability of the pitch within the analyzed voice sample |
| Acoustic Jitt (%) | Gives an evaluation of the variability of the pitch period within the analyzed voice sample in percent. It represents the relative period-to-period (very short-term) variability |
| Acoustic DDP | This is the average absolute difference between consecutive differences between consecutive periods, divided by the average period. |
| Acoustic NHR | Noise-to-harmonic ratio is an average ratio of energy of the in-harmonic components in the range 1500–4500 Hz to the harmonic components energy in the range 70–4500 Hz. It is a general evaluation of the noise present in the vocalization |
| Acoustic NUV | Number of unvoiced segments detected during the autocorrelation analysis |
| Acoustic NVB (%) | The number of distances between consecutive pulses that are longer than 1.25 divided by the pitch floor. |
| Acoustic PFR | Phonatory fundamental frequency range in semitones |
| Acoustic F0spread50to84 | Estimated spreading 84% to median |
| Acoustic F0spread16to50 | Estimated spreading median to 16% |
| Acoustic Mean_absolute_slope | Mean absolute slope of pitch |
| Acoustic PPQ (%) | Pitch perturbation quotient gives an evaluation in percent of the long-term variability of the pitch period within the analyzed voice sample at smoothing factor 3 periods |
| Acoustic RAP (%) | Relative average perturbation gives an evaluation of the variability of the pitch period within the analyzed voice sample at smoothing factor 3 periods |
| Acoustic Shdb (dB) | Shimmer in dB gives an evaluation in percent of the variability of the peak-to-peak amplitude within the analyzed voice sample |
| Acoustic APQ (%) | Amplitude perturbation quotient gives an evaluation in percent of the variability of the peak-to-peak amplitude within the analyzed voice sample at smoothing factor 11 periods |
| Acoustic Shimmer (apq3) | The three-point Amplitude Perturbation Quotient, the average absolute difference between the amplitude of a period and the average of the amplitudes of its neighbours, divided by the average amplitude. |
| Acoustic Shimmer (apq5) | The five-point Amplitude Perturbation Quotient, the average absolute difference between the amplitude of a period and the average of the amplitudes of it and its four closest neighbours, divided by the average amplitude. |
| Acoustic Shimmer (dda) | The average absolute difference between consecutive differences between the amplitudes of consecutive periods |
| Acoustic Shim (%) | Shimmer percent gives an evaluation in percent of the variability of the peak-to-peak amplitude within the analyzed voice sample. It represents the relative period-to-period (very short-term) variability of the peak-to-peak amplitude |
| Acoustic SPI | Soft phonation index is an average ratio of the lower-frequency to the higher-frequency harmonic energy. This index is not a measurement of abnormality but rather a measurement of the spectral ‘‘type’’ of the vocalization |
| Acoustic Tsam (sec) | Length in seconds of analyzed voice data sample |
| Acoustics voice breaks percentage | Fundamental frequency variation represents the relative standard deviation of the period-to-period calculated F0. It reflects the very long-term variations of F0 within the analyzed voice sample |
| Acoustic VTI | Voice turbulence index is an average ratio of the spectral in-harmonic high-frequency energy to the spectral harmonic energy in stable phonation areas. VTI measures the relative energy level of highfrequency noise, such as turbulence |
| Both Sides Total Content Units | Total correct content units both sides of picture |
| Both Sides Total Content Units Unique | Both Sides Total Content Units Unique |
| Count of content units on LEFT side of picture | Total correct content units left side of picture |
| Count of DISTINCT content units on Left side of picture | Count of DISTINCT content units on Left side of picture |
| Total Content Units / Total Words | Total number of Content Units / Total Number of Words |
| Count of content units on RIGHT side of picture | Total correct content units right side of picture |
| Count of DISTINCT content units on RIGHT side of picture | Total correct unique content units right side of picture |
| Count of ALL content units | Total number of Content Units |
| Count of ALL DISTINCT content units | Total unique number of Content Units |
| Count of RELATED content units | Total Related Content Units |
| Count of DISTINCT RELATED content units | Total Related Content Units Unique |
| Auxiliary Verbs | be, is, am, are, have, has, do, does, did, get, got, was, were |
| Conjunction | and, but, for, yet, neither, or, so, when, although, however, as, because, before |
| Definite Article | the |
| Demonstrative | this, that, those, these |
| Count of difference words: other,another | other, another |
| Count of distributive determiners | all, both, half, either, neither, each, every, none |
| Count of indefinite articles | a, an |
| Count of modals | may, might, can, could, will, would, shall, should, must, ought to |
| Numbers | (numbers) |
| Particles | up, down, off, to etc. |
| Possessive Determiners | their, your, my, his, her, its, our |
| Count of pre-determiners | such, what, rather, quite |
| Pro-sentences | yes, okay, ok, no, yeah |
| Qualifiers | very, really, quite, somewhat, rather, too, pretty, pretty much |
| Count of quantifiers | a few, a little, much, many, a lot of, most, some, any, enough, several, none, enough |
| Question Words | how, where, what, when, why, who |
| Total Function Words / Content Units | Total Function Words / Content Units |
| Total Function Words / Total Words | Total Function Words / Total Words |
| Count of all function words | Count of all function words |
| Count of complete sentences | Count of complete sentences |
| Syllables per word max | Syllables per word max |
| Syllables per word min | Syllables per word min |
| Number of Adverbs | Number of Adverbs |
| Count of nouns | Count of nouns |
| Count of phrases | Count of phrases |
| Count of prepositions | Count of prepositions |
| Count of pronouns | Count of pronouns |
| Count of sentences | Count of sentences |
| Count of Syllables | Count of Syllables |
| Count of utterances | Count of utterances |
| Number of Verbs | Number of Verbs |
| Count of words | Count of words |
| Syllables: Utterances Ratio | Syllables : Utterances Ratio |
| Syllables: Word Ratio (mean) | Syllables : Word Ratio (mean) |
| Variability (vector distance) | Variability (vector distance) |
| SD Number of Syllables / Word | SD Number of Syllables / Word |

# I. Receiver Operator Characteristic Curves for PD Risk Scores


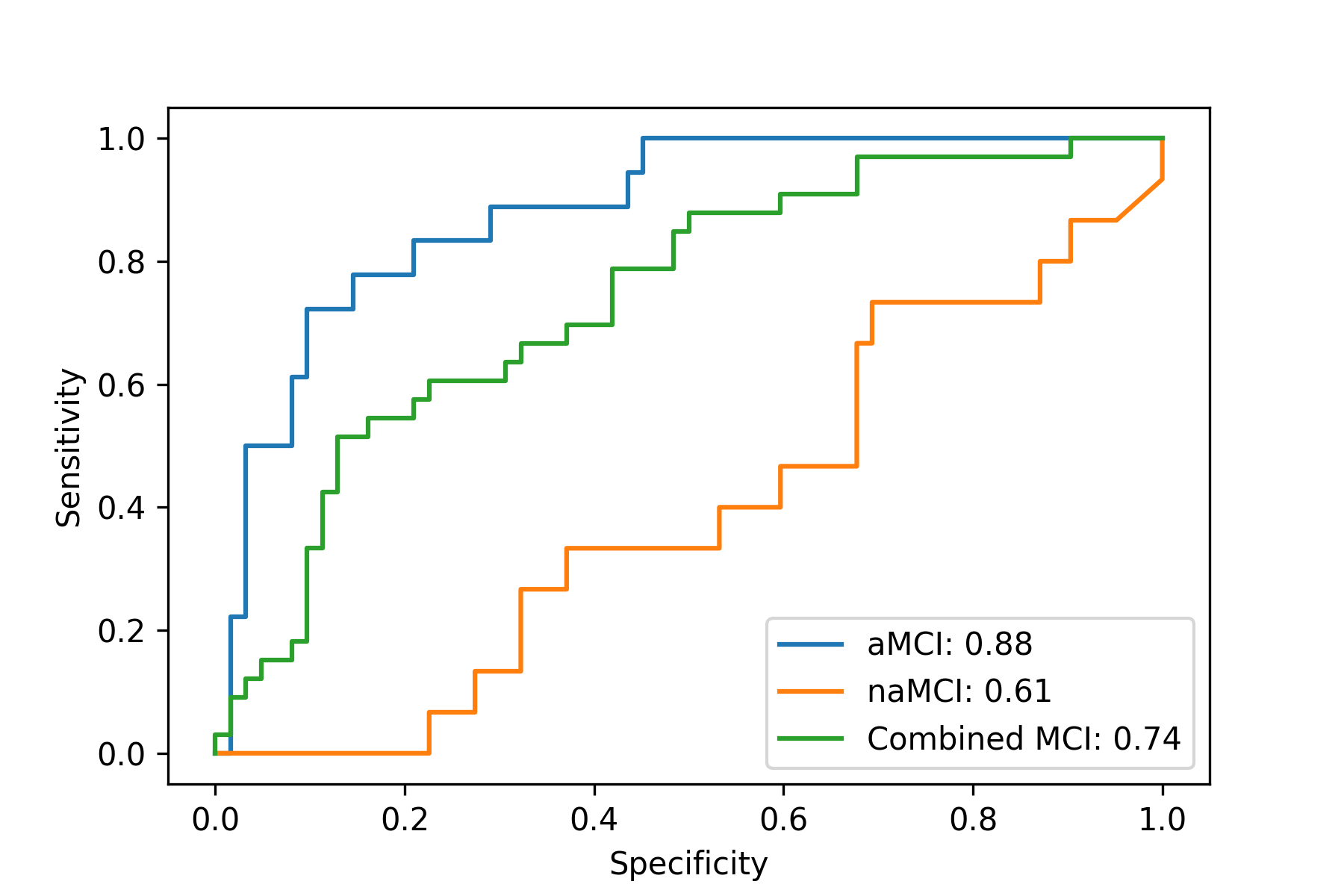


### Figure D. Receiver operator characteristic curves for PD Risk Scores used to distinguish healthy controls (HC) from either aMCI, naMCI or combined MCI cases

J. Data Security

Recordings are encrypted on the device (Apple iPad for this study) and sent in encrypted format to company HIPPA-compliant servers.

Recordings are processed to automatically extract acoustic features such as voice fundamental frequency.

No voice recordings or any other user data are sent to other companies’ servers for analysis. A proprietary speech-to-text system is used on-device to allow interactive games/ assessments that involve tasks like repeating sequences of numbers backwards. Recordings from picture description and interactive games are re-analyzed with proprietary speech-to-text systems when the recordings are uploaded to Miro’s secure HIPPA-compliant servers. For picture description responses in particular, we focus on transcripts made by trained transcriptionists.

There is a secure transcription application that trained transcriptionists working for Miro check to see if there are recordings awaiting transcription or transcriptions awaiting verification. They listen to recordings and type or annotate the transcriptions within the application. Transcripts are stored on the secure servers. Transcripts are automatically processed to extract language and content-unit features. There is no other way to access the recordings. Transcriptionists do not have access to any other information about the Miro Health Assessment Platform user.
